# Supplementary material for: Thiamine hydrochloride, riboflavin, pyridoxine hydrochloride, and biotin hard gelatin capsules prepared in advance and stored for the treatment of pediatric metabolic diseases: a safer alternative
Source: PLoS One. 2025 Apr 21;20(4):e0321136. doi: 10.1371/journal.pone.0321136 (PMC12011293; doi:10.1371/journal.pone.0321136)
Supplement: S1 Fig — Thiamine hydrochloride representative chromatograms. (DOCX) [file pone.0321136.s001.docx]

**Figures 1. Thiamine hydrochloride representative chromatograms**


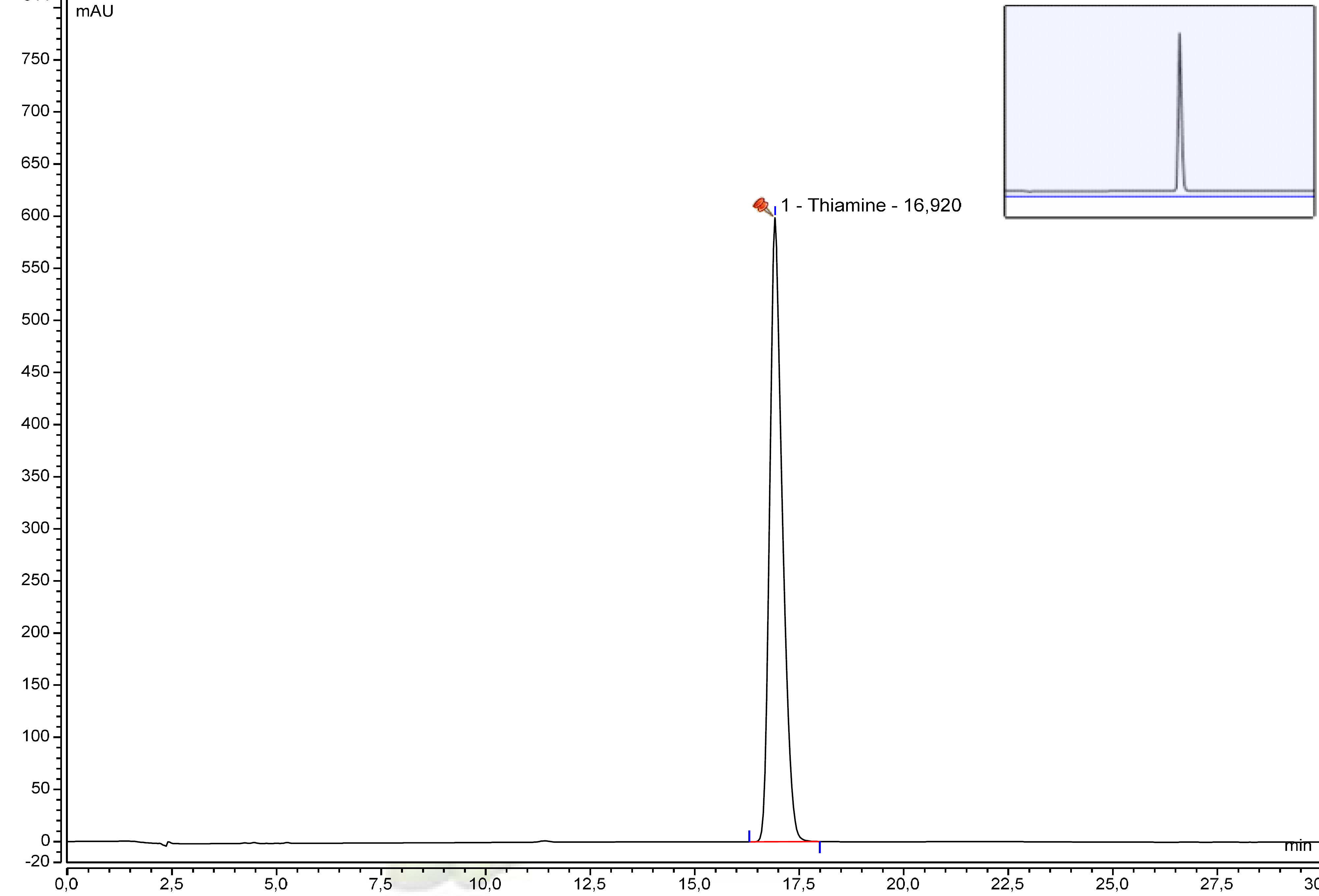


**Fig. 1.A. Thiamine hydrochloride, 250 µg.mL^-1^**


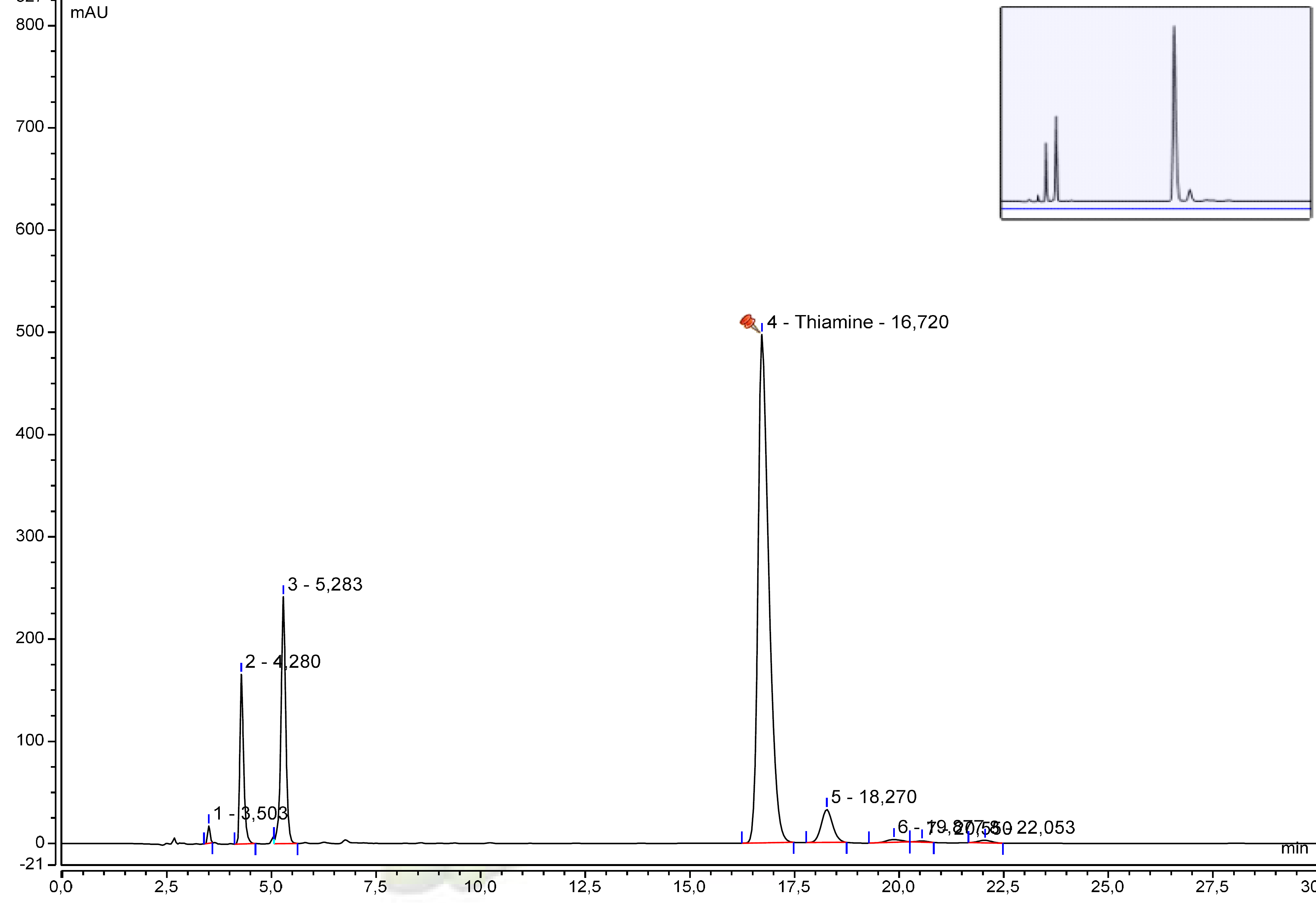


**Fig. 1.B. Heat: 80^o^C, 10 days**


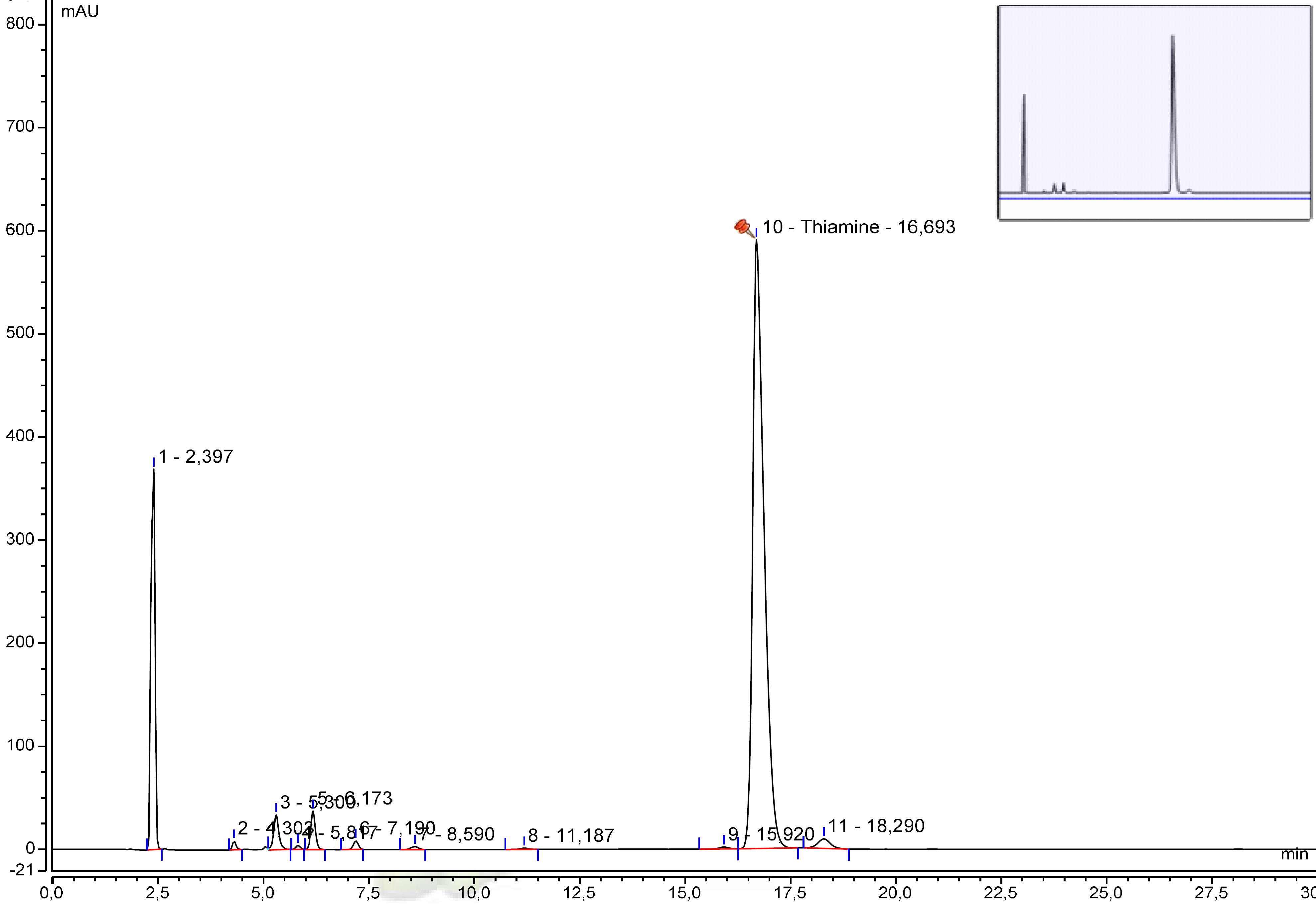


**Fig. 1.C. Oxidation: H_2_O_2_ 1.5%, 10 days**


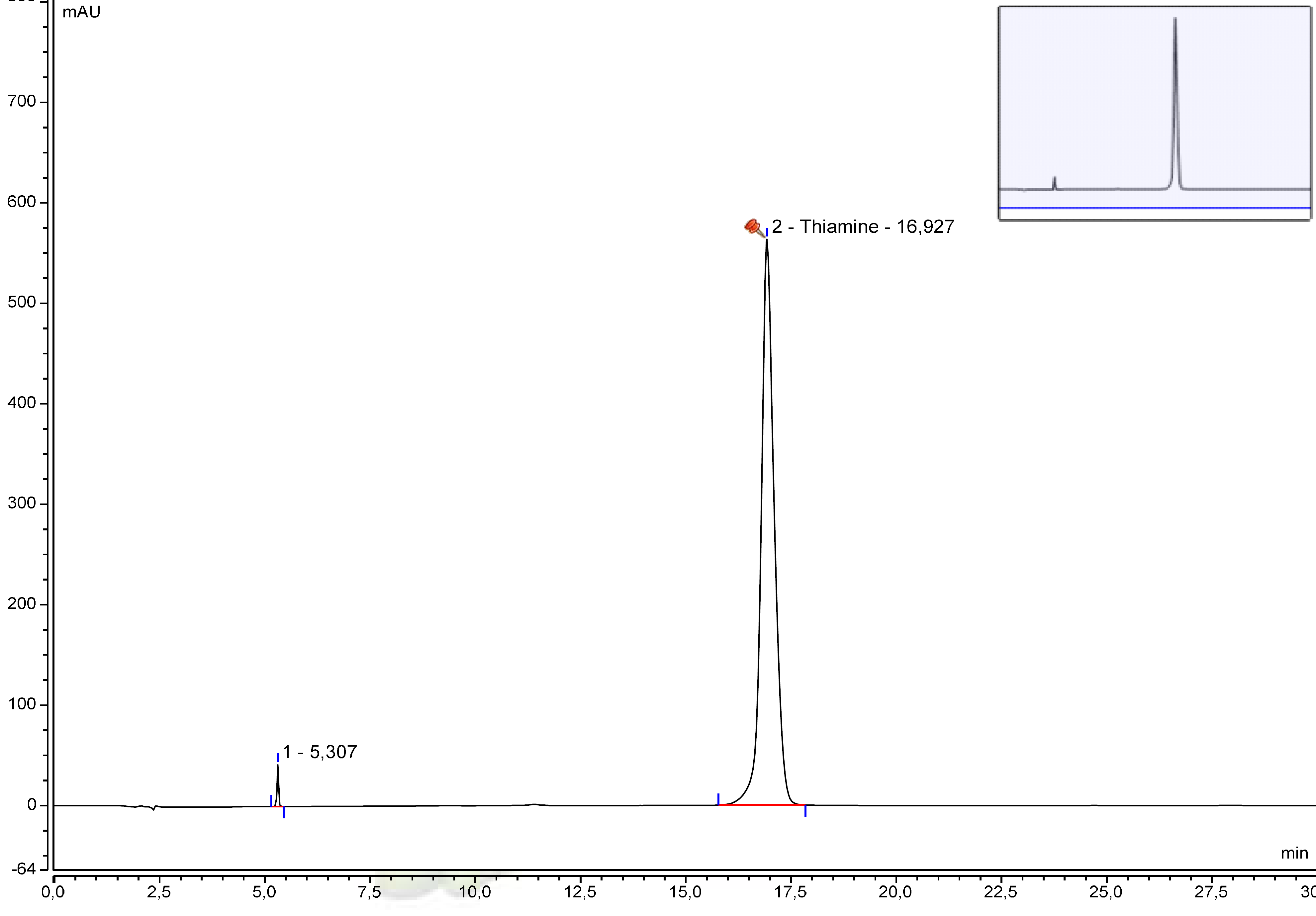


**Fig. 1.D. Acid: HCl 2 N, 20 days**


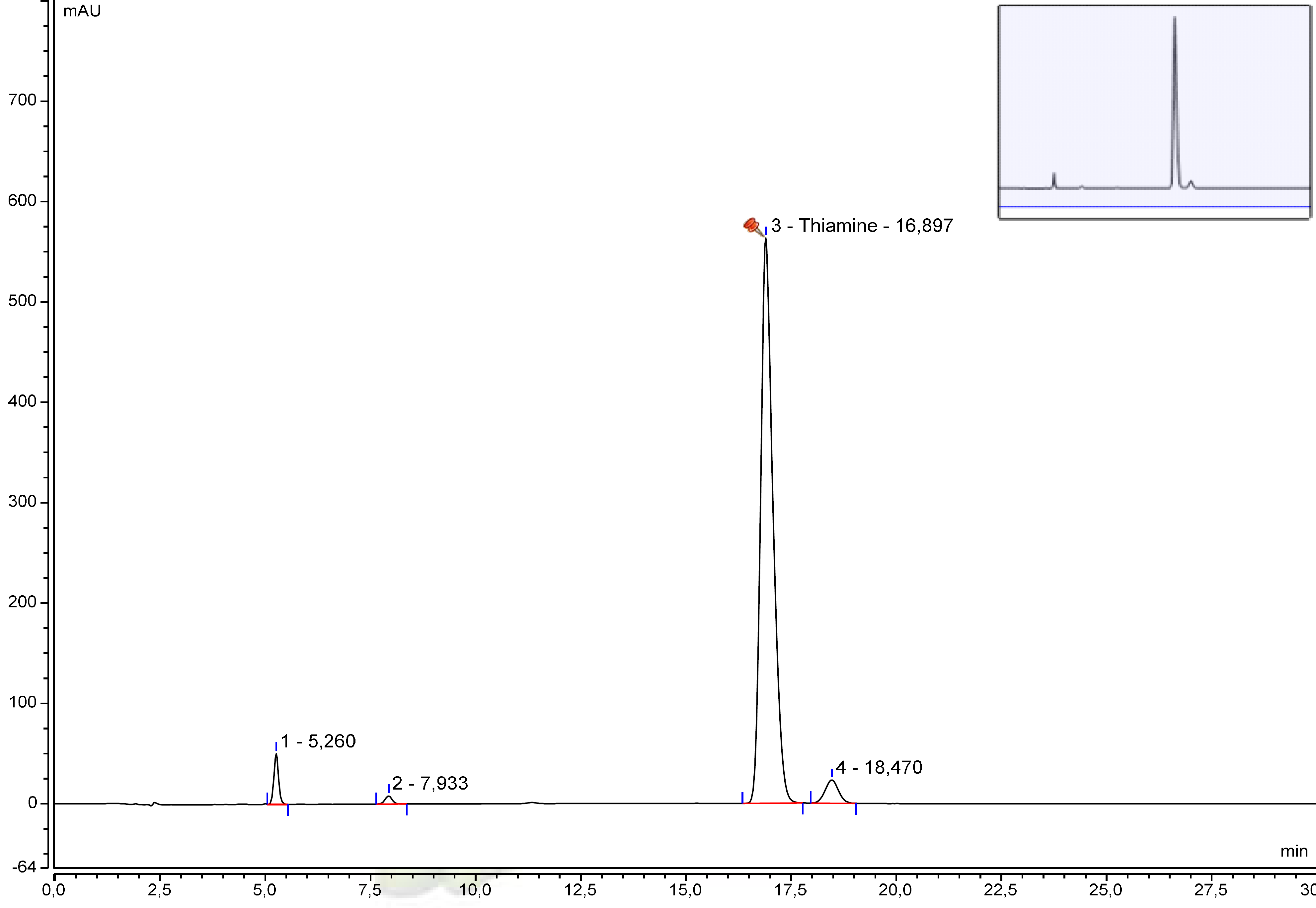


**Fig. 1.E. Alkaline : NaOH 0.5 M, 45 min**
