# Supplementary material for: Thiamine hydrochloride, riboflavin, pyridoxine hydrochloride, and biotin hard gelatin capsules prepared in advance and stored for the treatment of pediatric metabolic diseases: a safer alternative
Source: PLoS One. 2025 Apr 21;20(4):e0321136. doi: 10.1371/journal.pone.0321136 (PMC12011293; doi:10.1371/journal.pone.0321136)
Supplement: S2 Fig — Riboflavine representative chromatograms. (DOCX) [file pone.0321136.s002.docx]

**Figures 2. Riboflavine representative chromatograms**


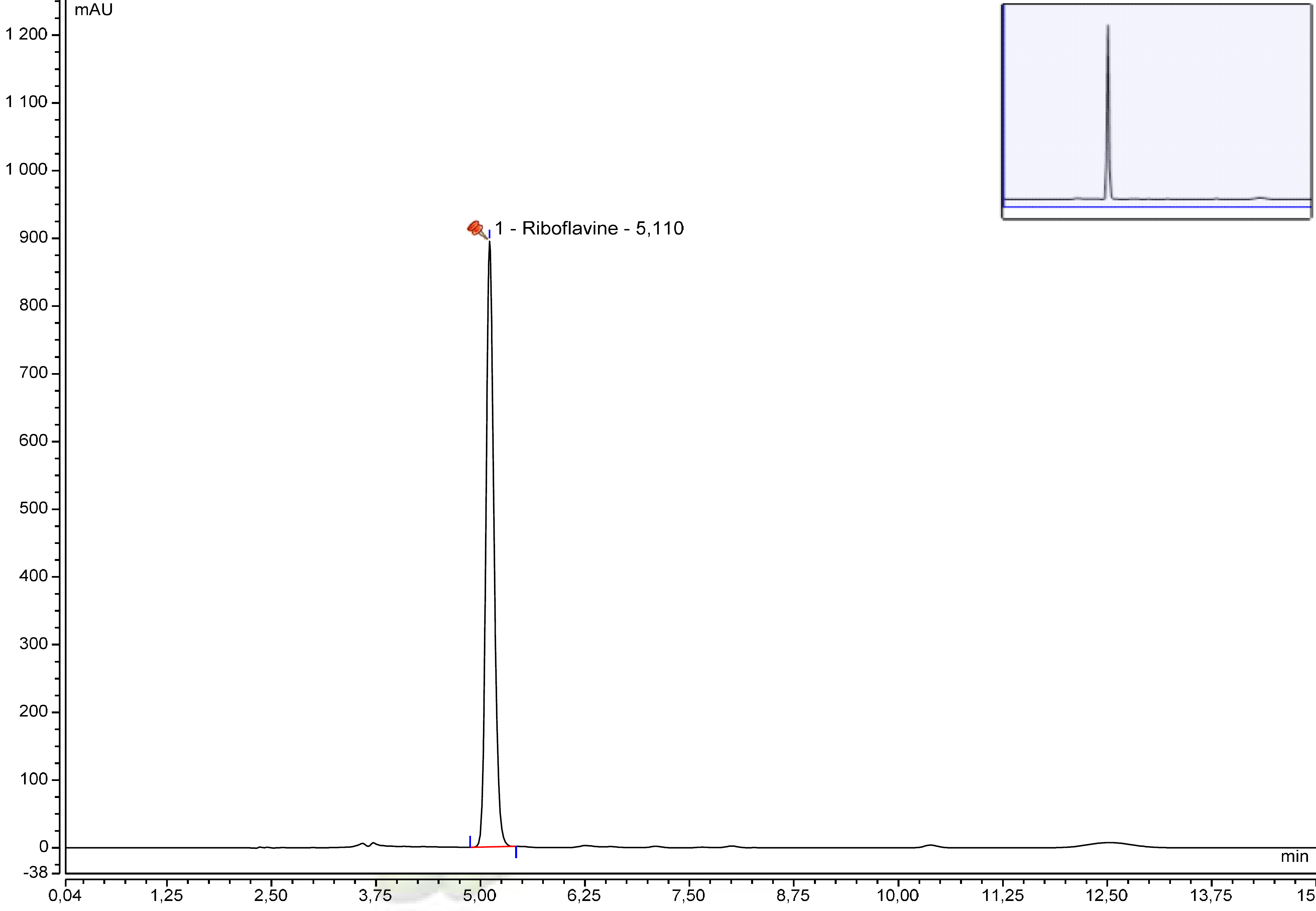


**Fig. 2.A. Riboflavine, 150 µg.mL^-1^**


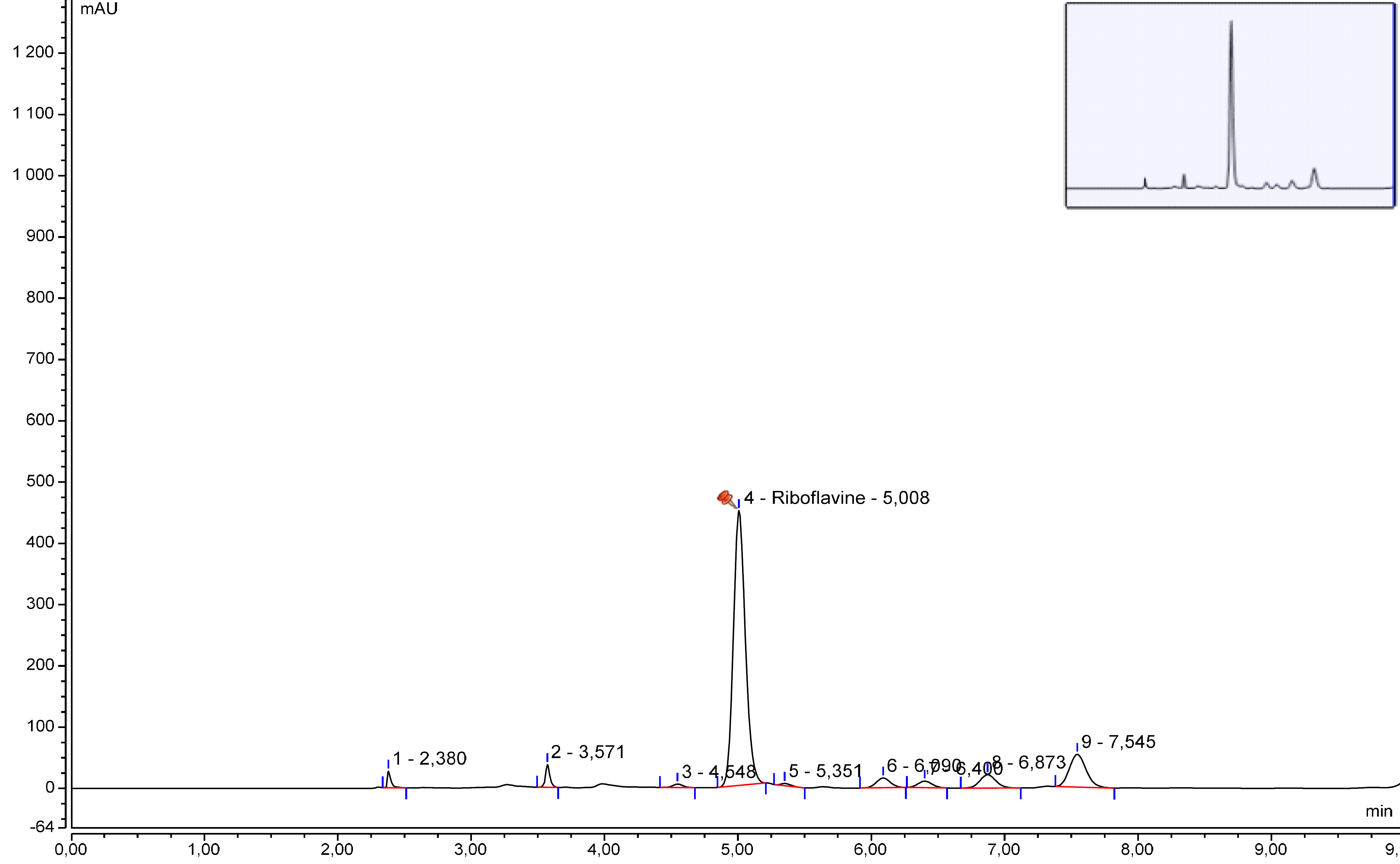


**Fig. 2.B. Heat: 50^o^C, 22 h**


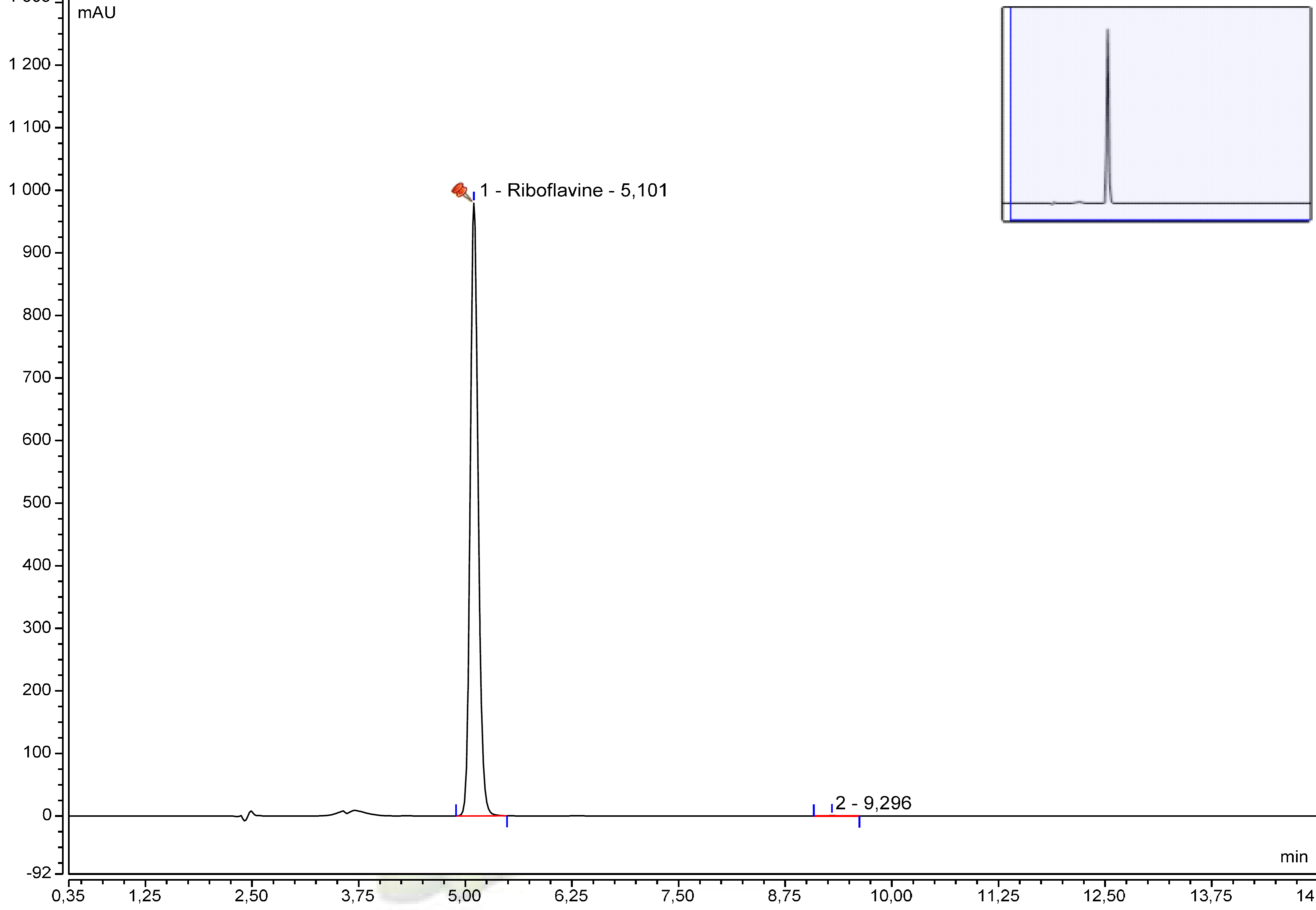


**Fig. 2.C. Acid: HCl 2 N, 24 h**


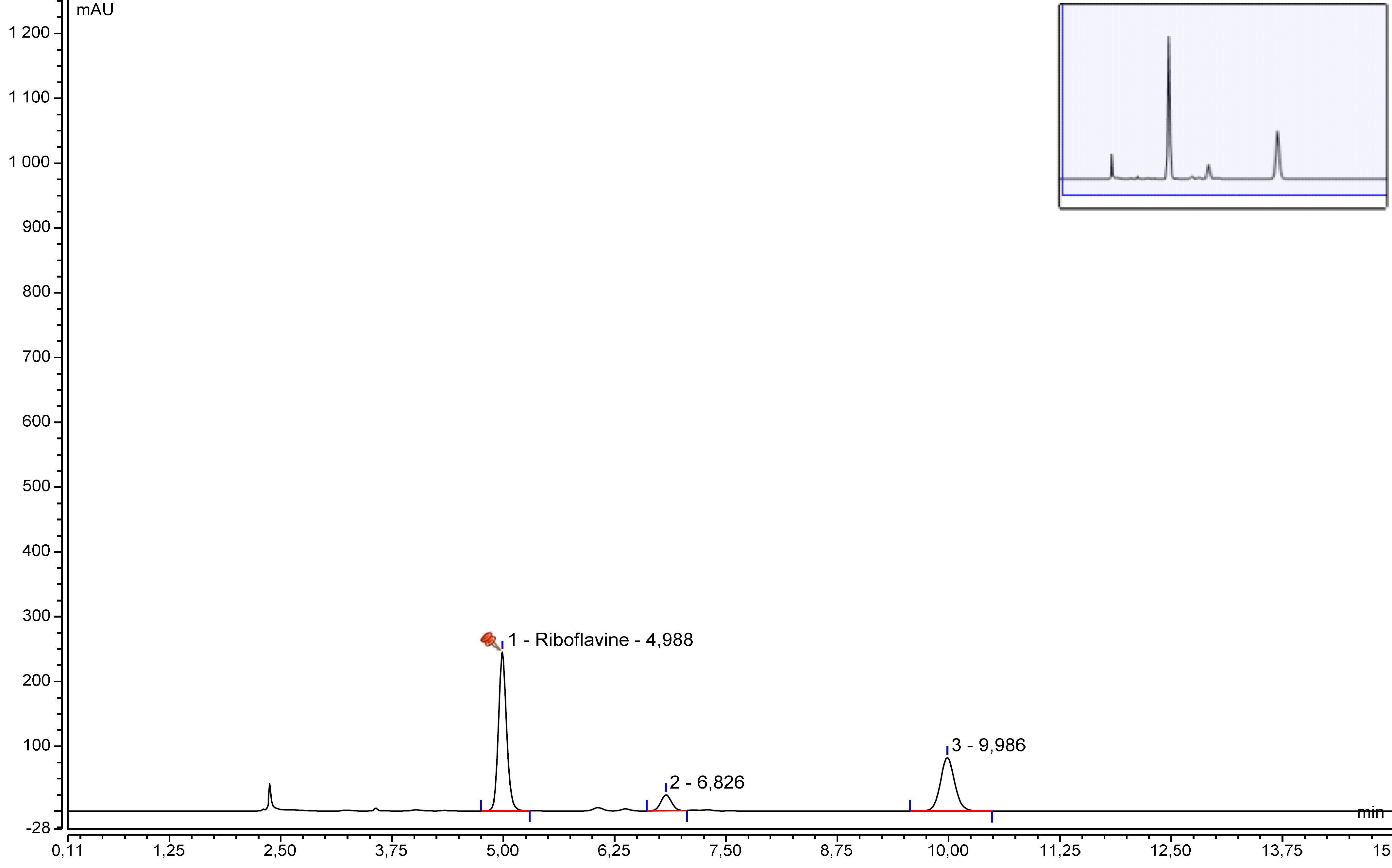


**Fig. 2.D. Alkaline: NaOH 1 M, 19 h**


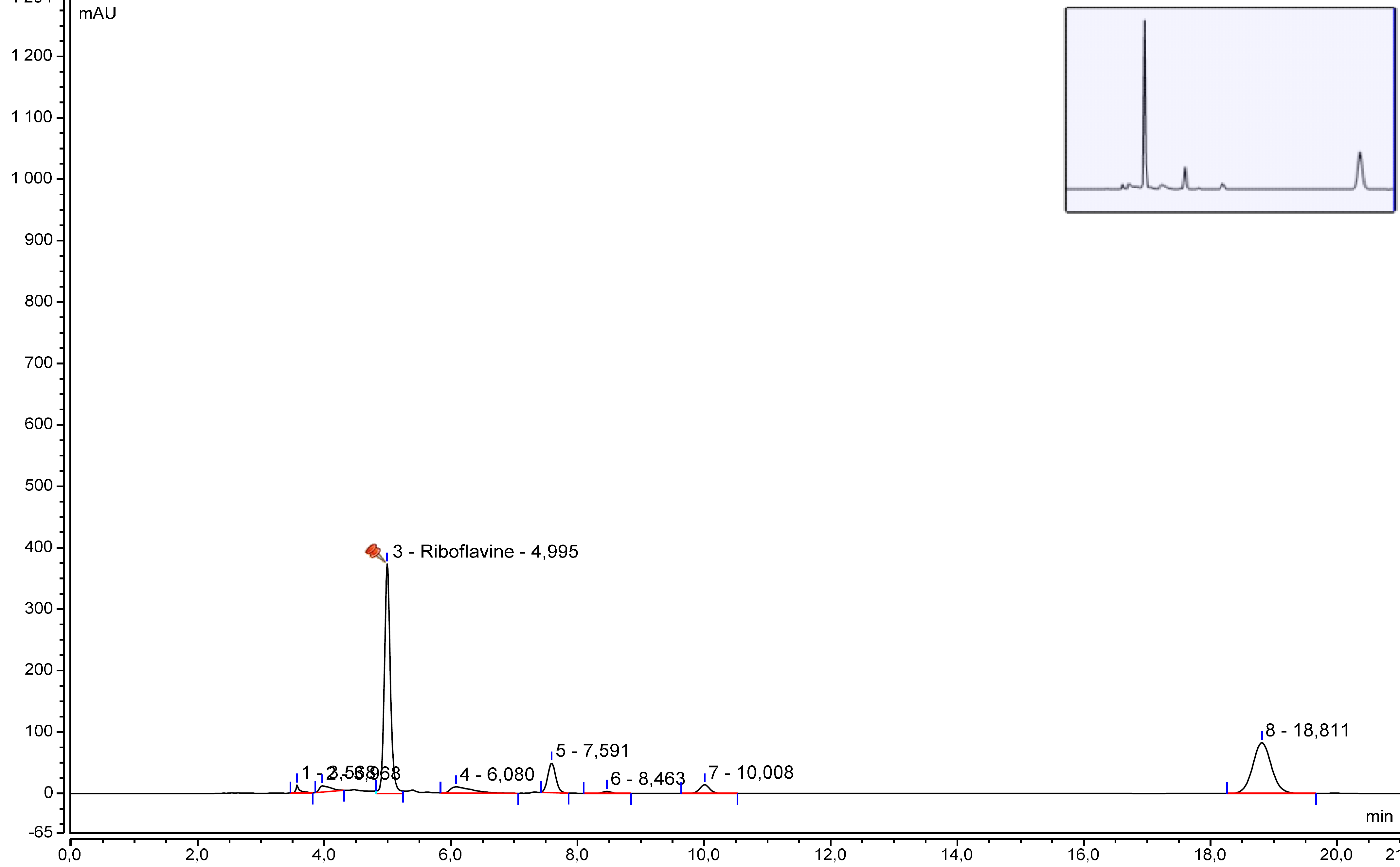


**Fig. 2.E. Light: 20 min**
