# Supplementary material for: Thiamine hydrochloride, riboflavin, pyridoxine hydrochloride, and biotin hard gelatin capsules prepared in advance and stored for the treatment of pediatric metabolic diseases: a safer alternative
Source: PLoS One. 2025 Apr 21;20(4):e0321136. doi: 10.1371/journal.pone.0321136 (PMC12011293; doi:10.1371/journal.pone.0321136)
Supplement: S3 Fig — Pyridoxine hydrochloride representative chromatograms. (DOCX) [file pone.0321136.s003.docx]

**Figures 3. Pyridoxine hydrochloride representative chromatograms**


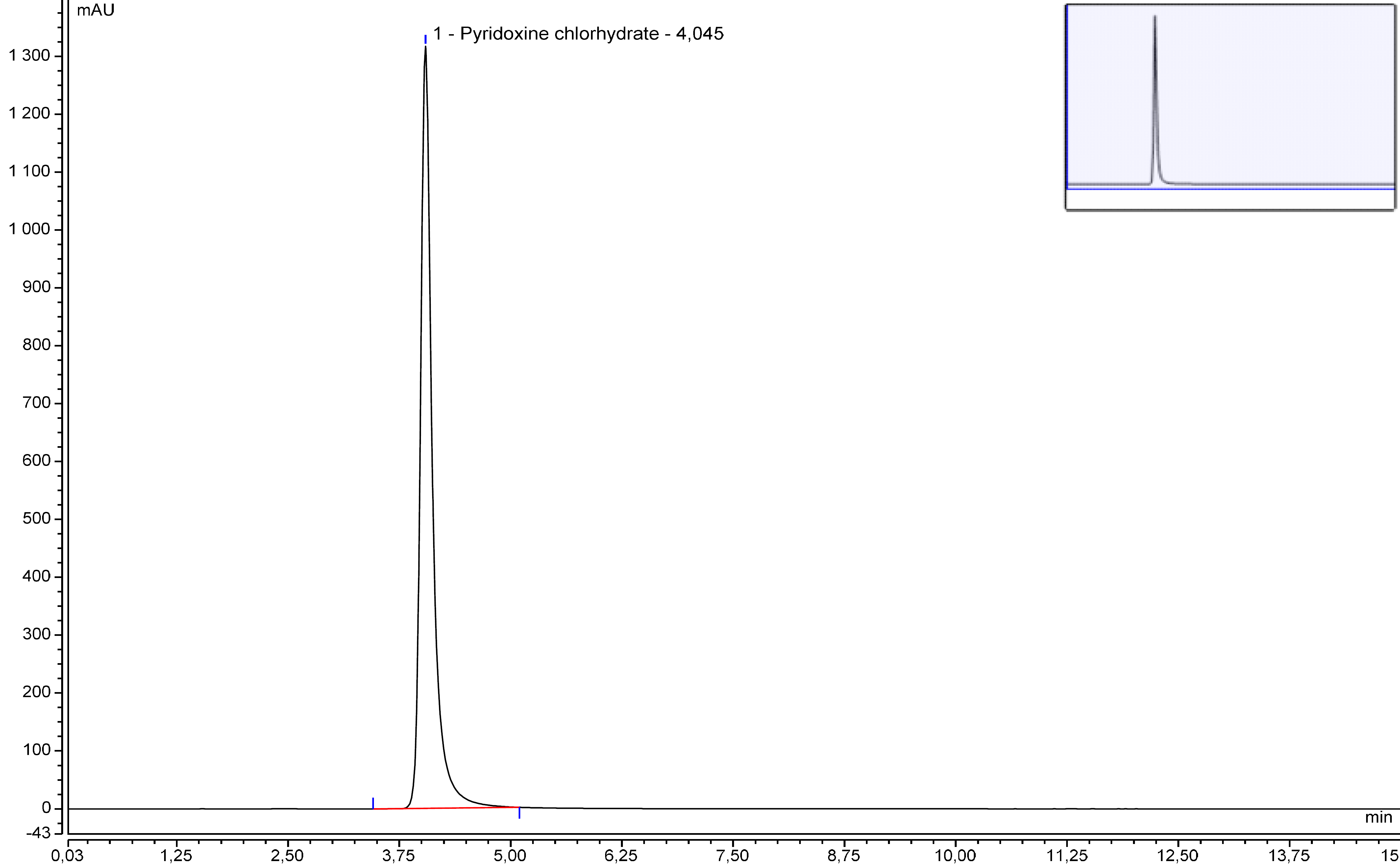


**Fig. 3.A. Pyridoxine hydrochloride, 500 µg.mL^-1^**


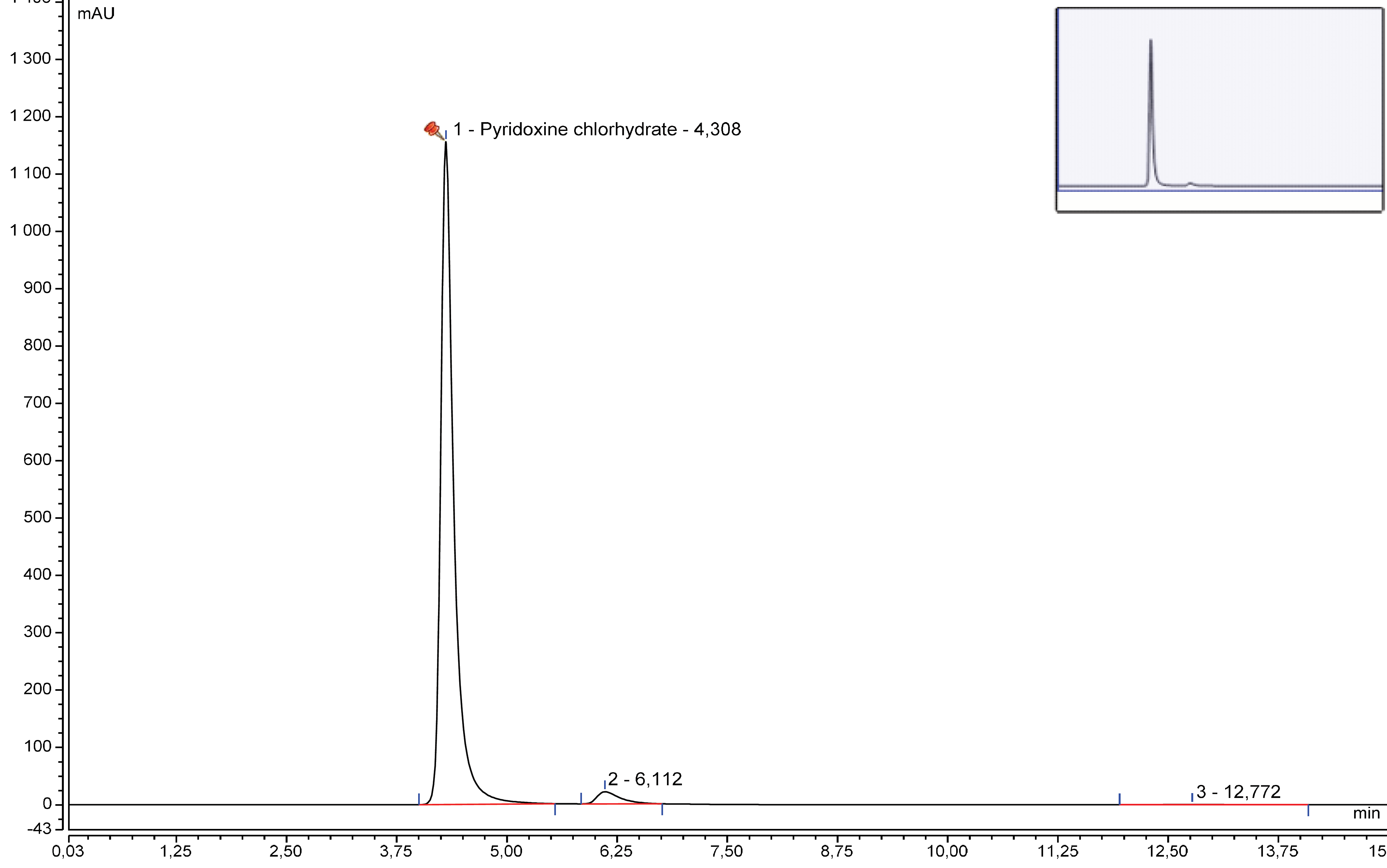


**Fig. 3.B. Heat: 50^o^C, 22 h**


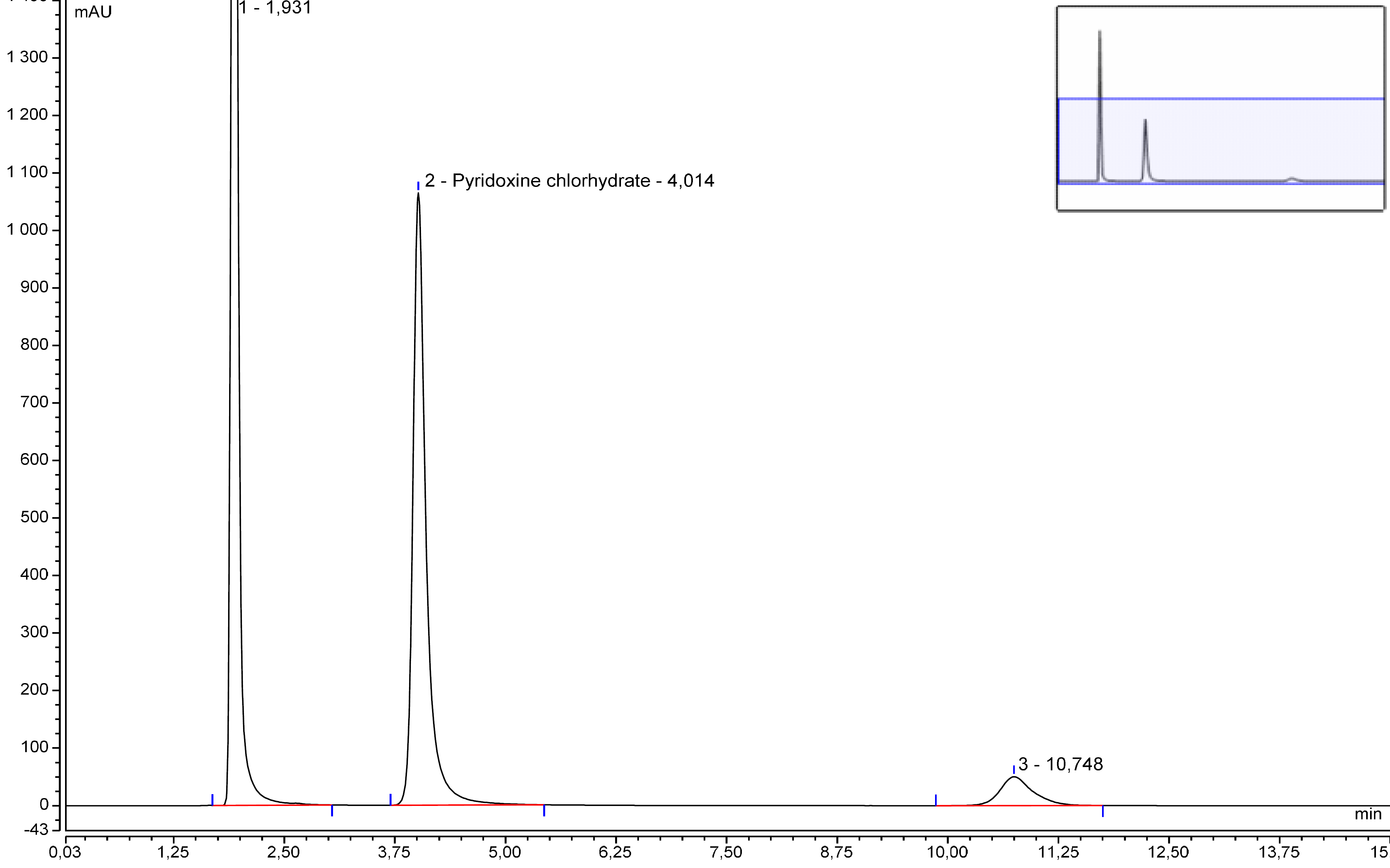


**Fig. 3.C. Oxidation: H_2_O_2_ 15%, 96 h**


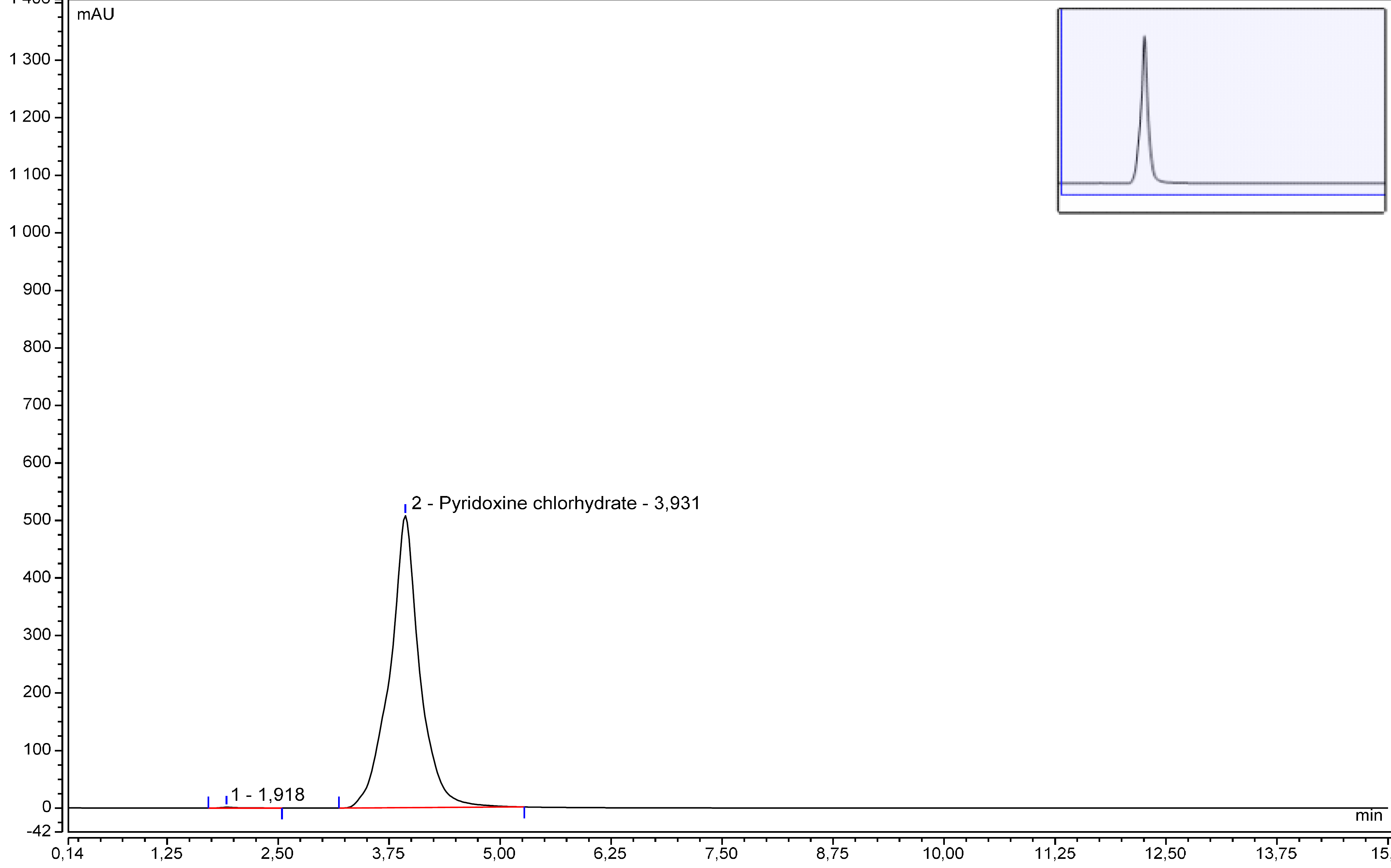


**Fig. 3.D. Alkaline: NaOH 3 N, 96 h**


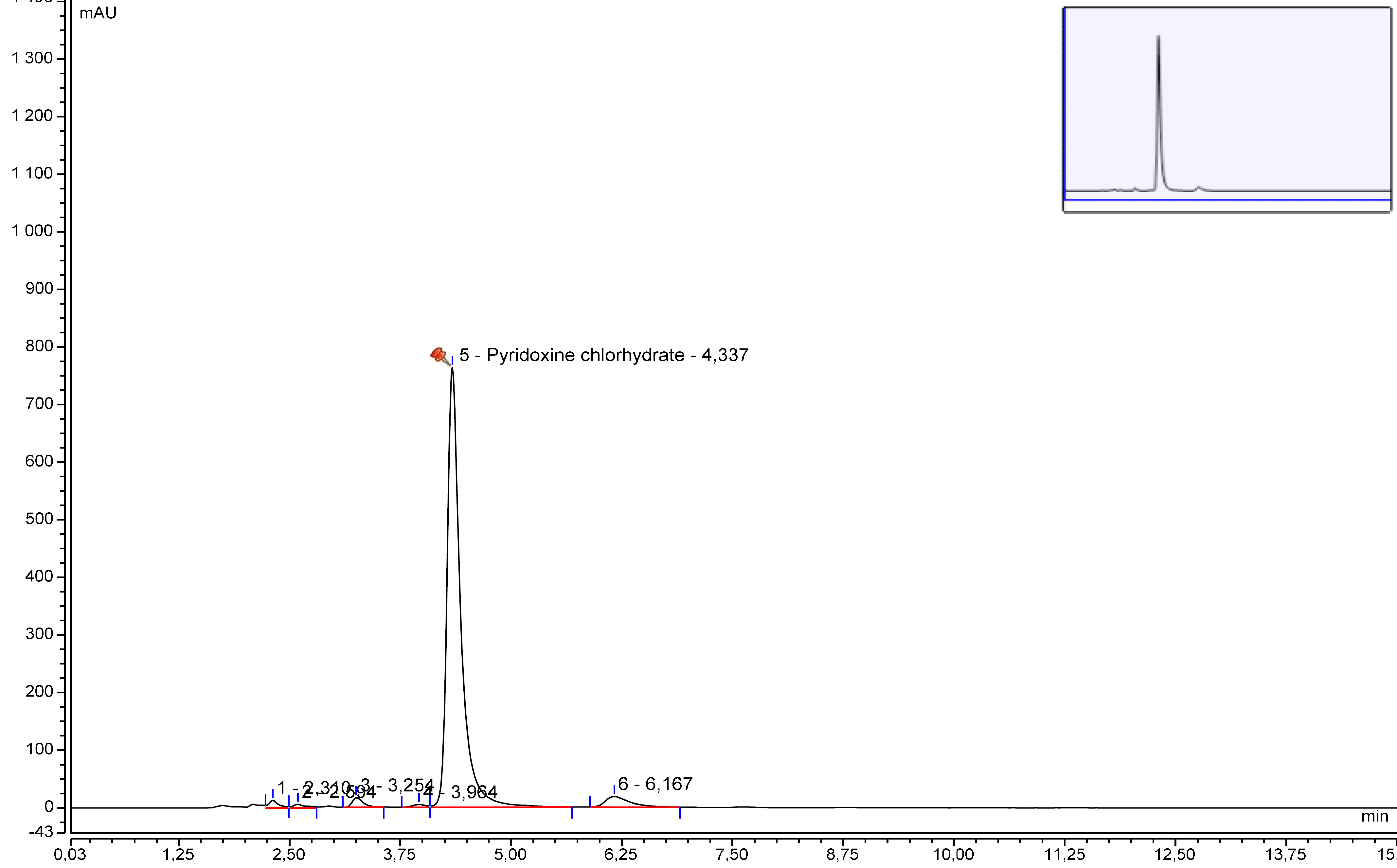


**Fig. 3.E. Light: 96 h**
