# Supplementary material for: Thiamine hydrochloride, riboflavin, pyridoxine hydrochloride, and biotin hard gelatin capsules prepared in advance and stored for the treatment of pediatric metabolic diseases: a safer alternative
Source: PLoS One. 2025 Apr 21;20(4):e0321136. doi: 10.1371/journal.pone.0321136 (PMC12011293; doi:10.1371/journal.pone.0321136)
Supplement: S4 Fig — Biotin representative chromatograms. (DOCX) [file pone.0321136.s004.docx]

**Figures 4. Biotin representative chromatograms**


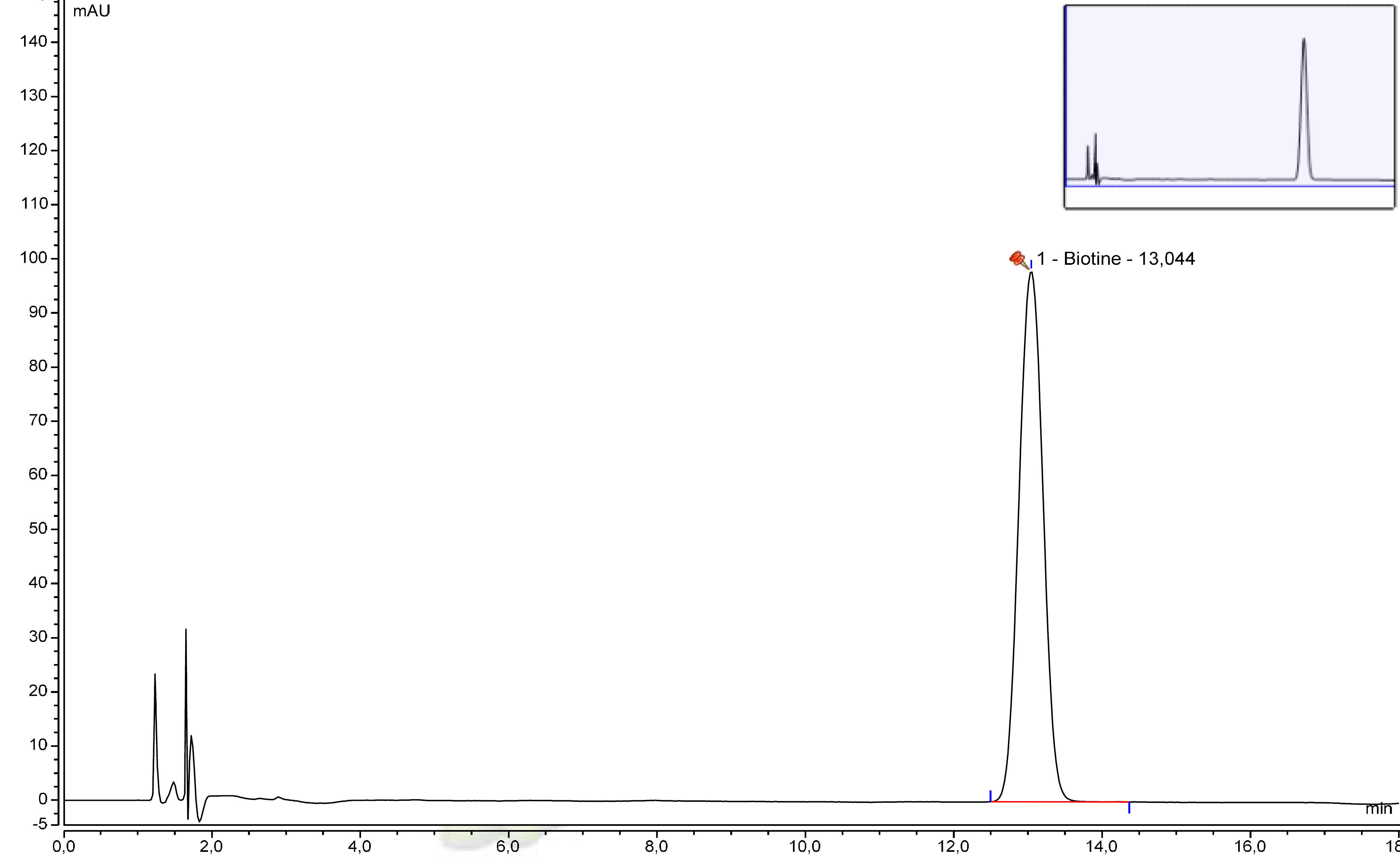


**Fig. 4.A. Biotin, 250 µg.mL^-1^**


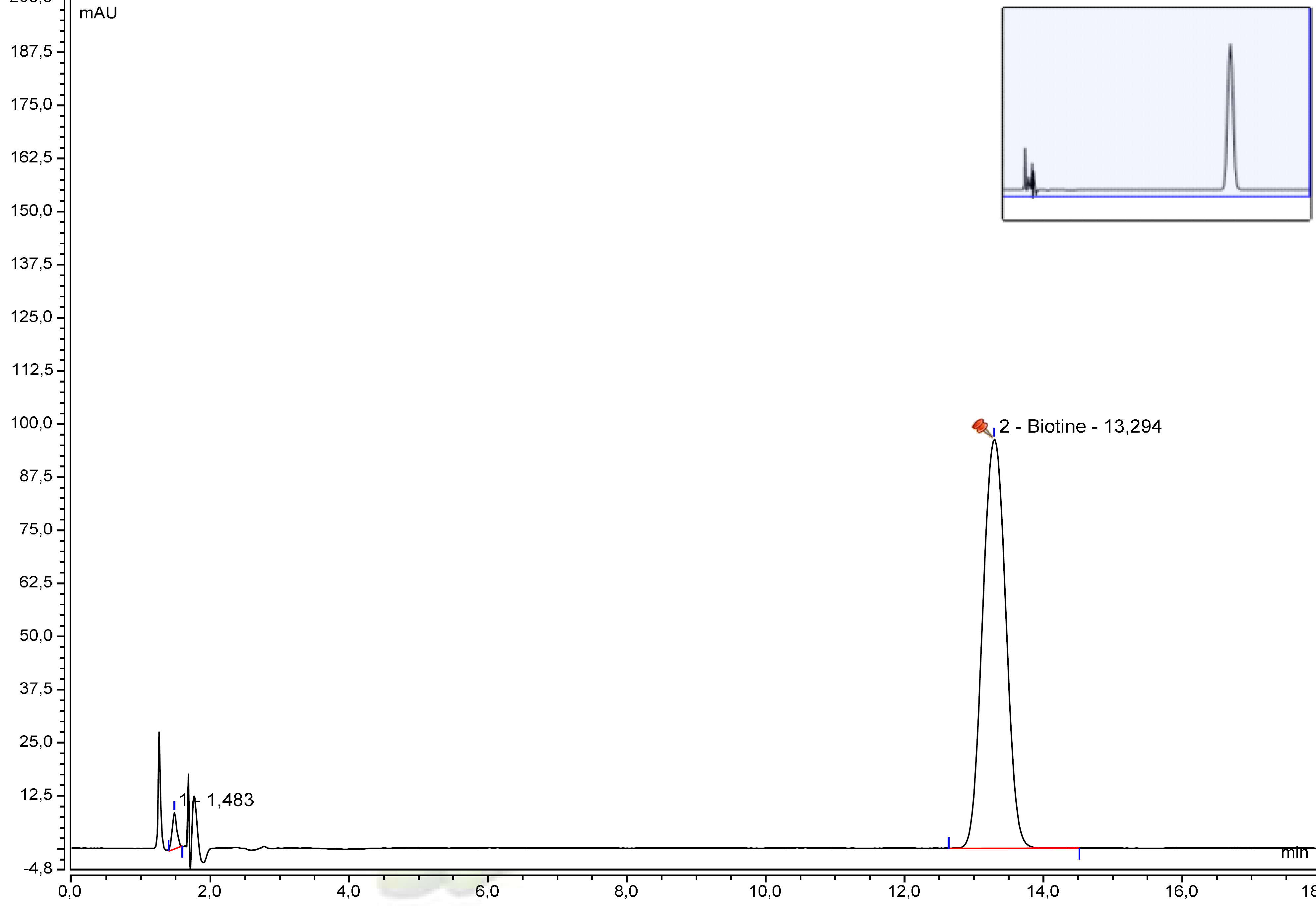


**Fig. 4.B. Heat: 80^o^C, 48 h**


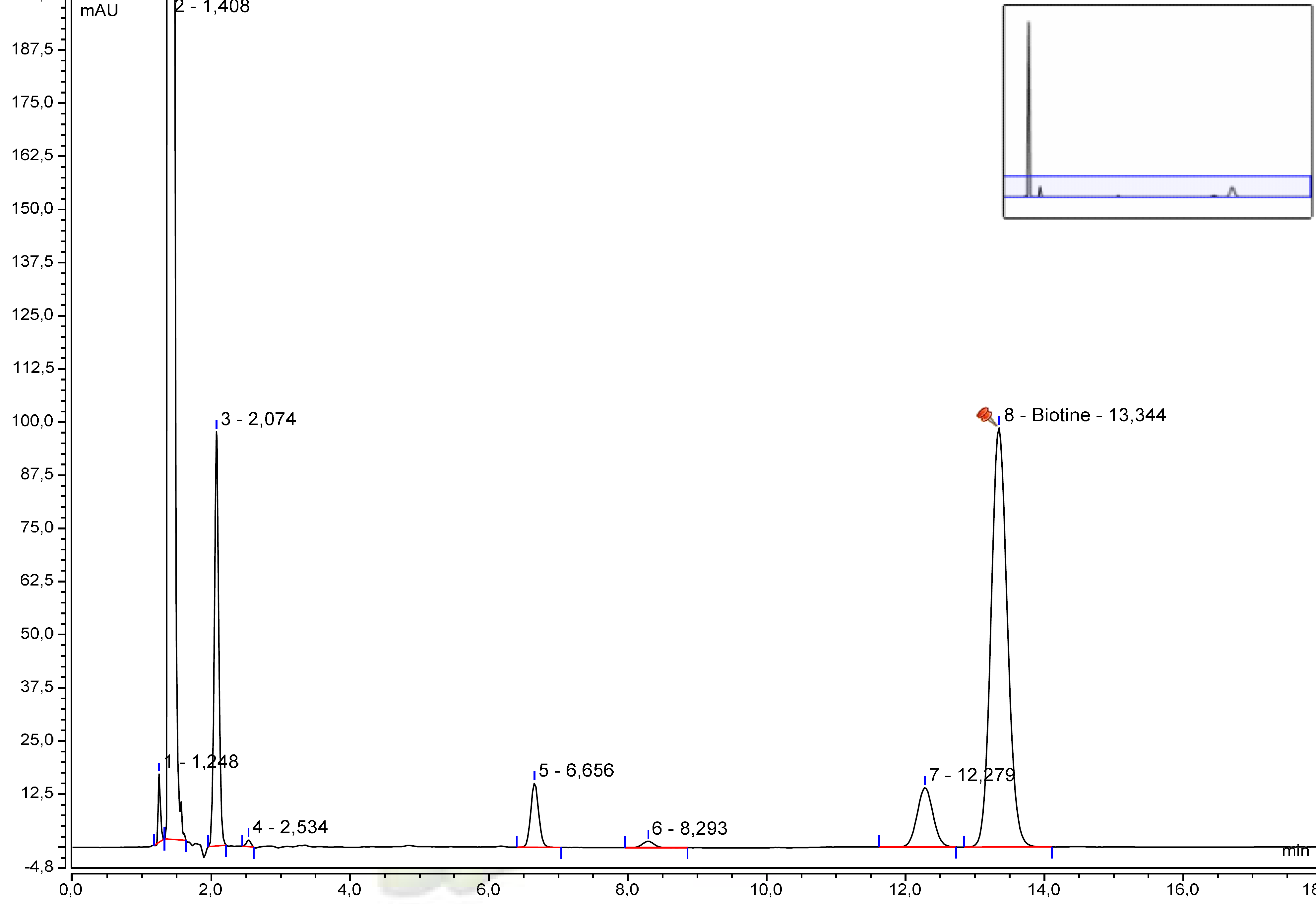


**Fig. 4.C. Oxidation: H_2_O_2_ 0.3%, 1 h**


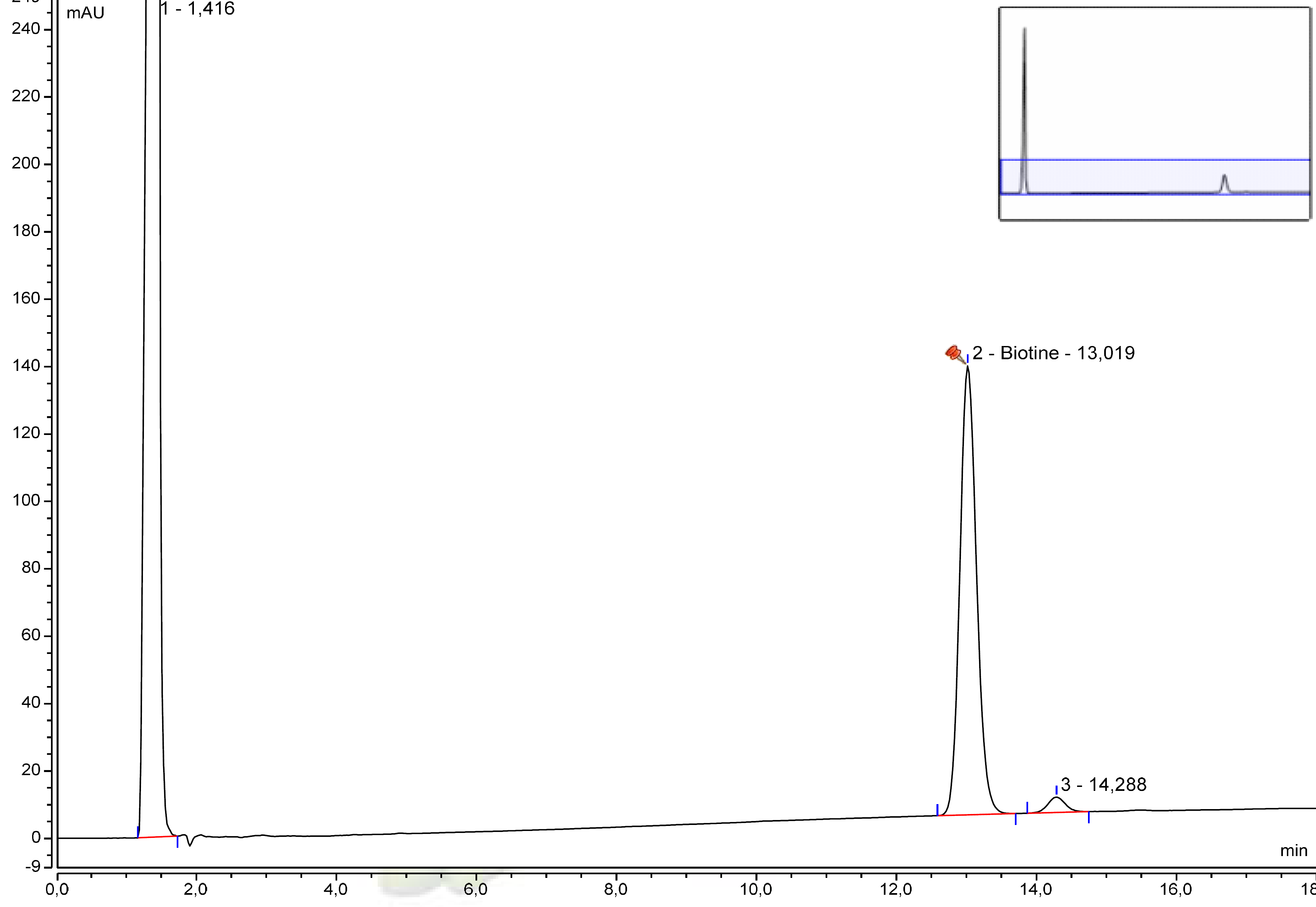


**Fig. 4.D. Acid: HCl 2 N, 2 h**


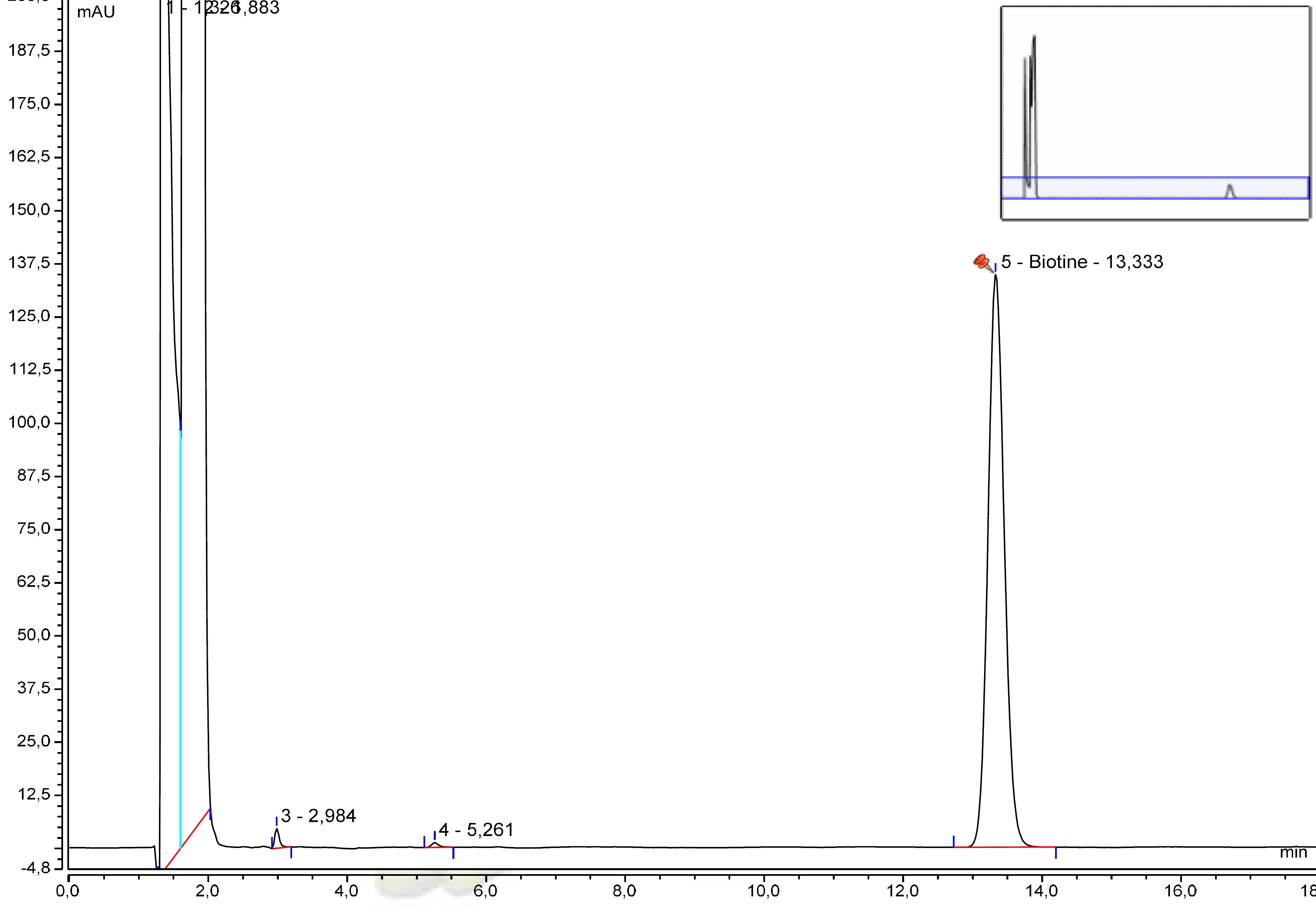


**Fig. 4.E. Alkaline: Saturated carbonates, 1 h**
