# Supplementary material for: Thiamine hydrochloride, riboflavin, pyridoxine hydrochloride, and biotin hard gelatin capsules prepared in advance and stored for the treatment of pediatric metabolic diseases: a safer alternative
Source: PLoS One. 2025 Apr 21;20(4):e0321136. doi: 10.1371/journal.pone.0321136 (PMC12011293; doi:10.1371/journal.pone.0321136)

**Figures 5. Content variations of vitamin capsules among time**

**Figure 5.A. Content variation of thiamine hydrochloride capsules stored at ambient temperature (protocol 1)**

**Figure 5.B. Content variation of riboflavin capsules stored at ambient temperature**

**Figure 5.C. Content variation of pyridoxine hydrochloride capsules stored at ambient temperature**


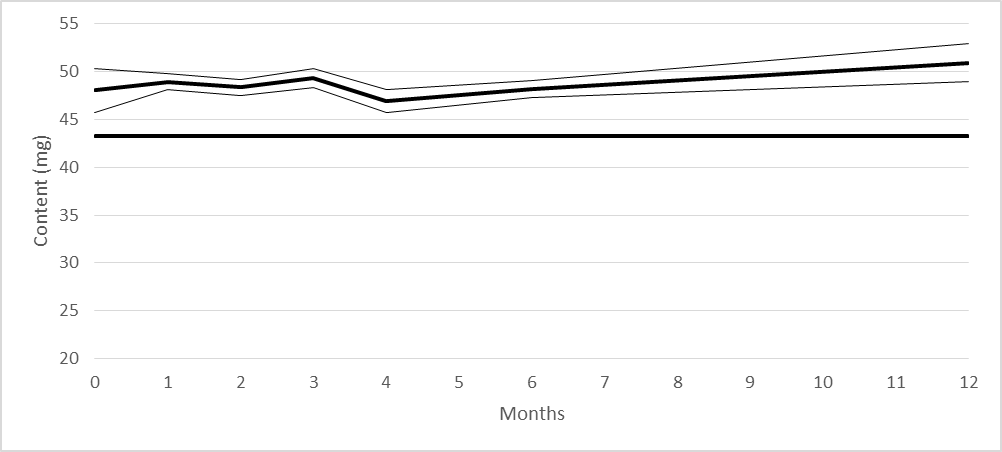


**Figure 5.D. Content variation of biotin capsules stored at ambient temperature**
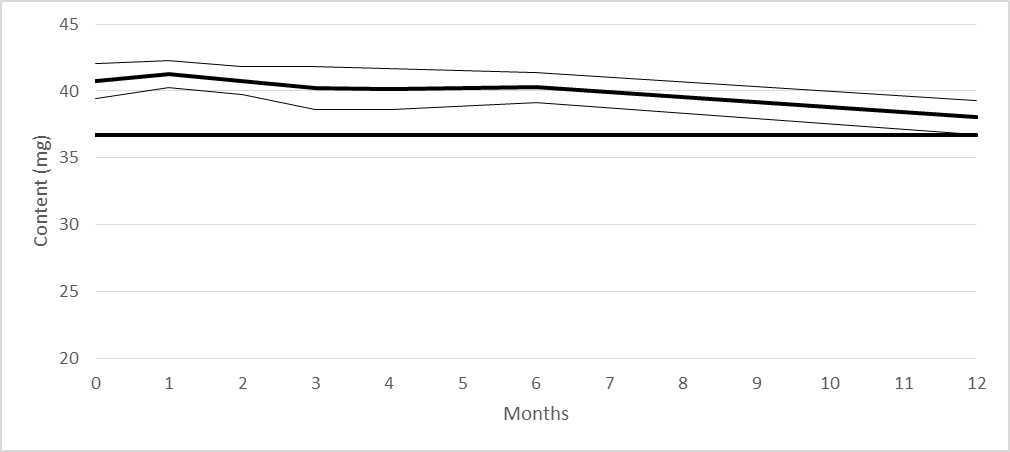

Supplement: S5 Fig — Content variations of vitamin capsules among time. (DOCX) [file pone.0321136.s005.docx]
