# Supplementary material for: Thiamine hydrochloride, riboflavin, pyridoxine hydrochloride, and biotin hard gelatin capsules prepared in advance and stored for the treatment of pediatric metabolic diseases: a safer alternative
Source: PLoS One. 2025 Apr 21;20(4):e0321136. doi: 10.1371/journal.pone.0321136 (PMC12011293; doi:10.1371/journal.pone.0321136)
Supplement: S1 Table — (DOCX) [file pone.0321136.s006.docx]

**Table 1. Bulk and tapped densities of APIs and excipients**

|  | **Bulk density (g.mL^-1^)** | **Tapped density (g.mL^-1^)** | **Theoretical weight for 300 capsules (size 4) / 63 mL (g)** | **Weight**  **for 300 capsules**  **(size 4) / 63 mL (g)** | **Diffe-rence** |
| --- | --- | --- | --- | --- | --- |
| Thiamine hydrochloride | 0.23 | 0.33 |  |  |  |
| Riboflavin | 0.27 | 0.32 |  |  |  |
| Pyridoxine hydrochloride | 0.45 | 0.56 |  |  |  |
| Biotin | 0.26 | 0.35 |  |  |  |
| MCC^a^ | 0.32 | 0.44 |  |  |  |
| Silica | 0.04 | 0.04 |  |  |  |
| MCC^a^/silica (5 g / 0.75 g) | 0.19 | 0.25 |  |  |  |
| MCC^a^/silica (12.5 g / 0.75 g) | 0.25 | 0.33 |  |  |  |
| Thiamine/MCC^a^/ silica (3/1/0.15) (w/w/w) | 0.29 | 0.33 | 20.79 | 20.75 | -0.2% |
| Riboflavin/MCC^a^ (1/1g) (w/w) | 0.35 | 0.48 | 30.24 | 30.00 | -0.8% |
| Pyridoxine hydrochloride/MCC^a^ (1.25/1) (w/w) | 0.38 | 0.43 | 27.09 | 27.00 | -0.3% |
| Biotin/MCC^a^/silica (0.96/1/0.06) (w/w/w) | 0.26 | 0.40 | 25.20 | 25.25 | +0.2% |

^a^ MCC = Microcrystalline cellulose
