# Supplementary material for: Thiamine hydrochloride, riboflavin, pyridoxine hydrochloride, and biotin hard gelatin capsules prepared in advance and stored for the treatment of pediatric metabolic diseases: a safer alternative
Source: PLoS One. 2025 Apr 21;20(4):e0321136. doi: 10.1371/journal.pone.0321136 (PMC12011293; doi:10.1371/journal.pone.0321136)
Supplement: S1 File — (DOCX) [file pone.0321136.s007.docx]

**Quality control for vitamin hard gelatin capsules**

1. Content

Hard gelatin capsule content is determined by HPLC-UV analysis. Conformity values come from the USP, from the closest pharmaceutical form monograph (capsules or tablets).

Thiamine hydrochloride: NLT 90.0% and NMT 110.0% of the labeled amount (50 mg) (USP “thiamine hydrochloride tablets” monograph)

Riboflavin: NLT 95.0% and NMT 115.0% of the labeled amount (50 mg) (USP “riboflavin tablets” monograph)

Pyridoxine hydrochloride: NLT 95.0% and NMT 115.0% of the labeled amount (50 mg) (USP “pyridoxine hydrochloride tablets” monograph)

Biotin: NLT 90.0% and NMT 110.0% of the labeled amount (40 mg) (USP “biotin capsules” monograph)

1. Uniformity of dosage units

As tested samples are capsules with dose or ratio drug/substance higher than 25 mg/25%. Uniformity of dosage units, weigh variation test is applied as per both EP and USP.

One capsule is analyzed for its content, and 10 are accurately weighed individually, and then emptied. The shells are accurately weighed individually. The acceptance value is calculated and has to be lower than 15%.

1. Microbiological conformity

For each batches, two individual capsules are analyzed to determine TAMC and TYMC, with the same protocol than detailed in the main manuscript.

Table A. presents the values obtained from the three first batches for each vitamin. All batches were conforms both for content and for uniformity of dosage units.

**Table 2. First batches, content and Uniformity of dosage units**

|  | Content (% of theoretical value) | Acceptance Value |
| --- | --- | --- |
| Thiamine hydrochloride (Batch 1) | 103.4 % | 11.6 % |
| Thiamine hydrochloride (Batch 2) | 100.2 % | 2.7 % |
| Thiamine hydrochloride (Batch 3) | 100.8 % | 13.5 % |
| Riboflavin (Batch 1) | 98.3 % | 7.7 % |
| Riboflavin (Batch 2) | 100.0 % | 5.7 % |
| Riboflavin (Batch 3) | 96.4 % | 9.1 % |
| Pyridoxine hydrochloride (Batch 1) | 98.9 % | 11.3 % |
| Pyridoxine hydrochloride (Batch 2) | 105.0 % | 10.6 % |
| Pyridoxine hydrochloride (Batch 3) | 98.5 % | 8.2 % |
| Biotin (Batch 1) | 98.1 % | 12.7 % |
| Biotin (Batch 2) | 99.2 % | 13.5 % |
| Biotin (Batch 3) | 101.1% | 13.2 % |
