# Supplementary material for: Thiamine hydrochloride, riboflavin, pyridoxine hydrochloride, and biotin hard gelatin capsules prepared in advance and stored for the treatment of pediatric metabolic diseases: a safer alternative
Source: PLoS One. 2025 Apr 21;20(4):e0321136. doi: 10.1371/journal.pone.0321136 (PMC12011293; doi:10.1371/journal.pone.0321136)
Supplement: Attachment — Submitted filename: Response to reviewers.docx [file pone.0321136.s009.docx]

Dear Editor and reviewers,

Thank you for the evaluation of our work. Please, find below a rebuttal letter that responds to each point raised by the reviewers.

**Reviewer #1:**

**Line 26: I don't believe that magistral practice is characterized by the absence of any quality control, it's just not mandatory. Please correct**

The sentence “, without any quality control,” was deleted

**Please merge first two paragraph in the Introduction section**

The first two paragraphs were merged.

**Please describe how to detect excipients with known effects in Table 1.**

While most excipients are considered inactive, some can have a known action or effect. In Europe, they must be declared on the label and package leaflet of the medicine. The EMA has established a list of “excipients with known effects”, which is very useful in compounding practice for pediatric patients.

L61. “can contain excipients with known effects” was replaced by “can contain excipients with known effects (as defined and listed by the European Medicine Agency^13^)”

A new reference was added:

European Medicine Agency. Excipients labelling. [Cited 2025 January 27]. Available from: <https://www.ema.europa.eu/en/human-regulatory-overview/marketing-authorisation/product-information-requirements/excipients-labelling>

**Methods: Why you used red carmine? Is it validated procedure for your API? Mixing one components do not mean mixing another one. Please provide information about mixing duration**

We thank the reviewer for this remark. We omitted the mixing duration, which is longer for thiamine (25 min) than for others (10 min).

A table (Table 2) and a paragraph summarizing the general compounding procedure (including mixing duration) replaced the text from L75 to L99

Red carmine is routinely used as a tracer dye, as a complement visual help (“homogenization tracer”), to ensure powder uniformity. We totally agree with the reviewer’s comment (Mixing one component does not mean mixing another one), but red carmine is only complementary to validated mixing times.

**Why silica din not used in the volume based method, as well as in riboflavine and pyridoxine capsules?**

In the discussion, L315, we added a short explanation paragraph:

“Then, vitamin hard gelatin capsules were compounded to have the simplest formulation, using only microcrystalline cellulose when possible. Silica was added to improve the powder flowability^32^ before capsule filling only when this parameter was identified as a limitation during the initial batches compounding (thiamine hydrochloride and biotin).”

One reference was added:

Majerová D, Kulaviak L, Růžička M, Štěpánek F, Zámostný P. Effect of colloidal silica on rheological properties of common pharmaceutical excipients. Eur J Pharm Biopharm. 2016; 106: 2-8.

**Why exactly 15 g of MCC was added? Do you have data about bulk and tapped density?**

Yes, the weight values were calculated from bulk and tapped densities. We obtained decimal values, but decided to slightly round these values (with a difference between the theoretical and rounded weights lower than 1%, but with the correct amount of API). For example, for pyridoxine capsules, the theoretical weight is 27.09 g, rounded to 27.00 g (15.00 g of pyridoxine and 12.00 g of cellulose). There is a difference of approximately 0.3% between the theoretical weight (from tapped density) and the rounded weight.

We had not added these data in the manuscript to not extend it too much, but it was a mistake as two reviewers made this remark. We made the following corrections:

“To determine the formula for each vitamin capsule, we first evaluated the bulk and tapped densities for each individual component and for selected mixtures, using the same protocol as previously described.^17^ Then, we calculated the theoretical weights to achieve a volume of 63 mL (300 hard gelatin capsules, size 4) with the appropriate amount of Active Pharmaceutical Ingredient (API). Finally, we slightly rounded these theoretical weights to obtain a simplified formula, ensuring differences between the theoretical weight and the rounded weight lower than 1%. The results are summarized in a table in the supporting information.”

A new reference “Wasilewski M, Curti C, Bouguergour C, Panuccio C, Thevin P, Primas N, Lamy E, Vanelle P. Paediatric capsule compounding in hospital practices: by weight or by volume? Eur J Hosp Pharm. 2023; 30: 363-366.” was added

“Table A. Bulk and tapped densities of APIs and excipients” was also added in Supporting information.

**I suggest creating a Table with the formulations contents in the method section**

A table (Table 2) and a paragraph summarizing the general compounding procedure replaced the text from L75 to L99

**Line 167: Which water do you used?**

L167: “(distilled water, Cooper)” was added

**Whether sinkers were used in the disintegration test?**

No, we sometimes use sinkers for the dissolution test but never for disintegration. For disintegration test, disks can be used as per USP, but we didn’t use them for the tests described in this work.

L167: “and without disks” was added

**Line 220: I do not understand first sentence**

We apologize for the lack of clarity.

L220: “Thiamine hydrochloride dosing method was found to be linear between 50 and 500 µg.mL^-1^, riboflavin between 100 and 200 µg.mL^-1^, pyridoxine hydrochloride between 200 and 800 µg.mL^-1^ and biotin between 100 and 500 µg.mL^-1^” was replaced by

“Linearity was demonstrated for all four methods: between 50 and 500 µg.mL^-1^ for thiamine hydrochloride, between 100 and 200 µg.mL^-1^ for riboflavin, between 200 and 800 µg.mL^-1^ for pyridoxine hydrochloride and between 100 and 500 µg.mL^-1^ for biotin.”

**Which guidelines you used for forced degradation studies?**

Protocols for forced degradation studies are not detailed in the ICH. However, we used Methodological guidelines for stability studies of hospital pharmaceutical preparations established by a French society (GERPAC). These guidelines are very close to the protocols and requirements described in two frequently cited publications on the subject:

Blessy M, Patel RD, Prajapati PN, Agrawal YK. Development of forced degradation and stability indicating studies of drugs-A review. J Pharm Anal. 2014; 4: 159-165.

Bakshi M, Singh S. Development of validated stability-indicating assay methods--critical review. J Pharm Biomed Anal. 2002; 28: 1011-1040.

We already cited these publications in our manuscript (L284)

**Which statistical test was applied for conclusion about stability?**

As suggested in “ICH Topic Q 1 E Evaluation of Stability Data”, a 95 percent confidence limit was calculated and used to determine stability.

“An appropriate approach to retest period or shelf life estimation is to analyze a quantitative attribute (e.g., assay, degradation products) by determining the earliest time at which the 95 percent confidence limit for the mean intersects the proposed acceptance criterion.”

However, to answer the reviewer’s request, we realized a statistical analysis (to study batch poolability and the statistical significance of drug’s content variations), and the results were added to the manuscript:

L218: “The statistical analysis of the change in measured content over time was conducted in accordance with the ICH Q1E guidelines. Specifically, batch poolability was confirmed through an analysis of covariance (ANCOVA) using a significance level of 0.25. Since time × batch interactions and the main effect of batch were non-significant for each of the formulations studied, all batches were deemed poolable. Then, the relationship between the variation in active ingredient content over 12 months and time was estimated for each formulation using regression analysis and the calculation of the coefficient of determination R^2^.” was added

L266: “For thiamine hydrochloride, riboflavin and pyridoxine hydrochloride, no statistically significant decrease in active ingredient content over time was found during a 12-month period. For biotin capsules, a significant reduction in content was observed after 12 months (p = 0.002). This decrease was not statistically significant during the first 6 months (p = 0.082).” was added

**Line 280: I do not understand what is dosing method in the context of HPLC**

L280 (and L30): “dosing” was replaced by “chromatographic”

**Please provide data about regulatory basis for compounding in advance**

In our country, compounding in advance is allowed (“hospital preparations”, versus “magistral preparations” which are extemporaneously compounded) and involves analytical quality control. As this quality control decreases the risk of error, hospital pharmacists try when possible to favor such “standardized” preparations. Moreover, as there was several compounding errors in pediatric practice due to extemporaneous compounding without control, our regulatory agencies encourage us to realize “standardized” and controlled preparations. However, as the definition and regulation of pharmaceutical compounding are not harmonized worldwide, we slightly modified our manuscript (without detailing our national regulatory rules).

L357: “Hard gelatin capsules compounding in advance secures the administration in pediatric practice. These capsules are not made as emergency” was replaced by: “However, compounding hard gelatin capsules in advance ensures proper administration in pediatric practice. The definition and regulation of pharmaceutical compounding are not harmonized worldwide, but in several countries, two categories of preparations exist: “extemporaneous preparations” and “stock preparations”.^41-42^ In the literature, there is no comparison of these two practices, but only “stock preparations” can be fully analytically controlled with destructive sampling.^43^ In the present work, capsules are not made as emergency”

Three references were added:

41. Dooms M, Carvalho M. Compounded medication for patients with rare diseases. Orphanet J Rare Dis. 2018; 13: 1.

42. Carvalho M, Almeida IF. The Role of Pharmaceutical Compounding in Promoting Medication Adherence. Pharmaceuticals (Basel). 2022; 15: 1091.

43. Uriel M, Marro D, Gómez Rincón C. An Adequate Pharmaceutical Quality System for Personalized Preparation. Pharmaceutics. 2023; 15: 800.

**Reviewer #2:**

**• Some results could be further interpreted. For instance, while degradation products of riboflavin are identified, their potential pharmacological relevance or toxicity is not addressed. Similarly, the implications of biotin degradation products, such as biotin sulfoxide, for the overall system are not explored.**

We agree with this remark and wish could do it, but public hospital structures do not have the resources for such studies. We added this point as a limitation of our work in the discussion.

L319: “In the pharmaceutical industry, when degradation products are identified and characterized, their pharmacological relevance and/or their toxicity can be studied. However, during small-scale compounding’s stability studies, hospital pharmacists do not have the resources to perform such studies. This is one of the limitations of our study, but also more generally of every published stability study, attempting that the pharmaceutical industry rarely publishes its data.” was added.

**• The text could benefit from more concise language. Some instances of grammatical errors and awkward phrasing, such as "yielded to 22% degradation," can be improved for better readability.**

L300: “yielded to 22% degradation” was modified to “degraded pyridoxine (22% degradation rate)”.

Several slight modifications and table adjunctions were done to improve readability.

**• Line 49 – 50. For clarity, consider revising the sentence “Although the parenteral route is often prescribed as an emergency treatment both for thiamine3 and pyridoxine, the oral route of administration must be privileged as soon as possible.” to “While the parenteral route is commonly used for emergency treatment with thiamine and pyridoxine, the oral route should be prioritized as soon as feasible.”**

L49-50: “Although the parenteral … must be privileged as soon as possible.” Was replaced by “While the parenteral route is commonly used for emergency treatment with thiamine and pyridoxine, the oral route should be prioritized as soon as feasible.”

**• Line 105, insert the word “rate” between the words “at” and “a” in the fragment “…(VWR)) at a flow of 1 mL.min-1”**

These sentences were deleted and replaced by a synthetic table

**• Line 112. Replace the word “weighted with “weighed”
• This problem persists through the methods – correct all**

“weighted” was replaced by “weighed” in the whole manuscript

**• Line 354 – 356. For better clarity, rewrite the sentence “When capsules are poured in 2 mL of water, the obtained suspension must be fully administered and gently mixed when it is taken before administering to avoid any risk of underdosing.” The fragment “.. the obtained suspension must be fully administered and gently mixed when it is taken before administering to avoid any risk of underdosing” is difficult to understand. A suggested version is “When capsules are dissolved in 2 mL of water, the resulting suspension should be gently mixed and fully administered to ensure proper dosing and avoid any risk of underdosing.”**

L354: “When capsules are poured in 2 mL of water, the obtained suspension must be fully administered and gently mixed when it is taken before administering to avoid any risk of underdosing.” Was replaced by “When capsules are opened in 2 mL of water, the resulting suspension should be gently mixed and fully administered to ensure proper dosing and avoid any risk of underdosing.”

**• Consider revising the sentence “However, hard gelatin capsules compounding in advance secures the administration in pediatric practice.” in line 357 to read as: "However, compounding hard gelatin capsules in advance ensures proper administration in pediatric practice."**

L357: “hard gelatin capsules compounding in advance secures the administration in pediatric practice. These capsules are not made as emergency” was replaced by: “However, compounding hard gelatin capsules in advance ensures proper administration in pediatric practice.”

**Reviewer #3:**

**A significant flaw in the manuscript is the lack of results regarding the 'uniformity of dosage units' assay, which is a critical test as per the European and US Pharmacopeia guidelines. Uniformity of dosage units is essential to ensure that each capsule contains the correct amount of active ingredient. Although it is likely that the authors performed this assay, the manuscript does not present any data on weight uniformity or a full pharmacopeial assay on drug content.
This omission is a major concern because without this data, it is difficult to assess whether the observed variability in the stability results stems from inconsistencies in the compounding process itself or from factors unrelated to formulation stability. In the absence of dosage uniformity data, the article lacks the necessary information to fully evaluate the consistency of the compounded capsules, which could ultimately affect their safety and efficacy in a clinical setting.**

We did not include these results in our manuscript to decrease its length. It was a mistake.

All our hospital preparations are analytically and microbiologically controlled for the following parameters:

- Content
- Content uniformity. For capsules with a dose or a ratio drug/substance higher than 25 mg/25%, uniformity of dosage units (weigh variation test) is applied as per both EP and USP. One capsule is analyzed for its content, and 10 are accurately weighed individually, emptied and the shells are accurately weighed individually. The acceptance value is calculated and has to be lower than 15%.
- Microbiological contamination (TAMC and TYMC)

These data were summarized and added to the supporting Information. A table with the content and uniformity of dosage unit results from the first three batches released was also added to answer to reviewer’s request.

L244: “Routine quality control and results from the first three batches analyzed are summarized in the supporting information.” was added.

**Introduction section:**
**1- Neonates and Hard Capsules: The authors mention neonates in the context of the study, but hard capsules are generally not considered an appropriate dosage form for this age group due to issues such as swallowing difficulties and the need for precise dosing. It would be helpful if the authors could provide further explanation or justification for why hard capsules need to be compounded in this case, particularly for neonates. Are there specific circumstances or clinical reasons that warrant this formulation, or could alternative dosage forms (e.g., liquids or powders) be more suitable for this population?**

We thank the reviewer for this remark, as we omitted to detail this point.

L66, a paragraph of explanation was added:

“Therefore, these treatments must be compounded by the hospital pharmacists. In pediatric practice, liquid and solid oral formulations both have advantages and inconveniences. Solid forms do not need to be swallowed whole and should be dissolved in a small volume of liquid before administration. The advantages of hard gelatin capsules over oral liquids include improved stability, good dosage uniformity, lower cost and simplicity of their formulation. This simplicity also contributes to better safety, for instance through the absence of any preservatives, and improves the access to age-appropriate essential medicines for young children, particularly in low- and middle-income countries.^14-15^”

14. Orubu ES, Tuleu C. Medicines for children: flexible solid oral formulations. Bull World Health Organ. 2017 Mar 1;95(3):238-240. doi: 10.2471/BLT.16.171967.

15. World Health Organization. TRS 970 - Annex 5: Development of paediatric medicines: points to consider in formulation. Annex 5, WHO Technical Report Series 970, 2012. [Cited 2025 January 24]. Available from: https://www.who.int/publications/m/item/trs970-annex-5-development-of-paediatric-medicines-points-to-consider-in-formulation

**2- Table 1 - Terminology: In Table 1, the term ‘commercial drugs’ could be more accurately described as ‘available commercial formulations’ or ‘available commercial drug products’. This would provide a clearer and more precise reference. Additionally, it would be beneficial to specify which country or region this information pertains to, as pharmaceutical formulations can vary significantly by location.**

“commercial drugs” was replaced by “available commercial formulations” in Table 1, and also at L59 and L63.

L59: several commercial specialties are available was replaced by several commercial specialties are available in our country (France)

**3- Scope of the Article: The statement ‘In our hospital, prescribers…’ refers to a very specific context, which may limit the broader applicability of the article's findings. It would be helpful if the authors could clarify that the scope of the study extends beyond this specific setting. Ideally, the manuscript should aim to describe a problem or solution that is of general relevance to the wider hospital pharmacy community, rather than being confined to a single institutional context.**

We completely agree with this remark. However, since there are no commercially available dosages for these vitamins for the treatment of pediatric metabolic diseases and no clear galenic recommendations, the nominal dosages (50 mg for pyridoxine, thiamine, riboflavin and 40 mg for biotin) were established from the literature data (pediatric dosage regiment) and our prescribers’s experience (Reference center for pediatric genetic metabolic disorders).

Therefore, we can only recommend these dosages.

Moreover, in pediatric compounding practice, there are two different approaches: liquid oral formulations or solid oral formulations. Both have inconveniences and advantages (a short paragraph about this point was added in the introduction).

Therefore, we can only recommend solid oral formulations for the treatment of pediatric metabolic diseases.

L66: “In our hospital, prescribers asked pharmacists for hard capsules compounding” was replaced by “For the treatment of pediatric metabolic diseases, hard capsules can be compounded”

**Methodology section:**

**4- Capsules Compounding: Please provide the exact weights of the powders used in the study, including the number of decimals that reflect the precision of the weighing scale. This will ensure greater transparency and reproducibility of the compounding process.**

We wanted to obtain formulas with integer weighting values if possible. The values described are those we use in routine practice (15 g of cellulose = 15.00 g etc…).

The weight values were calculated from bulk and tapped densities. We obtained decimal values, but we decided to slightly round these values (ensuring a difference between the theoretical and the rounded weights lower than 1%, while maintaining the correct amount of API). For example, for pyridoxine capsules, the theoretical weight is 27.09 g, rounded to 27.00 g (15.00 g of pyridoxine and 12.00 g of cellulose). There is a difference of approximately 0.3% between the theoretical weight (derived from tapped density) and the rounded weight.

We had not added these data in the manuscript to not extend it too much, but it was a mistake as two reviewers made this remark. We made the following corrections:

“To determine the formula for each vitamin capsule, we first evaluated the bulk and tapped densities for each individual component and for selected mixtures, using the same protocol as previously described.^17^ Then, we calculated the theoretical weights to achieve a volume of 63 mL (300 hard gelatin capsules, size 4) with the appropriate amount of Active Pharmaceutical Ingredient (API). Finally, we slightly rounded these theoretical weights to obtain a simplified formula, ensuring differences between the theoretical weight and the rounded weight lower than 1%. The results are summarized in a table in the supporting information.”

A new reference “Wasilewski M, Curti C, Bouguergour C, Panuccio C, Thevin P, Primas N, Lamy E, Vanelle P. Paediatric capsule compounding in hospital practices: by weight or by volume? Eur J Hosp Pharm. 2023; 30: 363-366.” was added

“Table A. Bulk and tapped densities of APIs and excipients” was also added in Supporting information.

**4- Capsules Compounding: Additionally, this section could be streamlined by describing the general compounding procedure briefly, followed by a table that presents the specific weightings for each formulation. This would make the methodology more concise and easier to follow.**

A table (Table 2) and a paragraph summarizing the general compounding procedure replaced the text from L75 to L99.

**5- HPLC Stability-Indicating Method: The description of the HPLC method could be more concise. This streamlined information will provide a clearer and more focused explanation without unnecessary repetition. Please include the following key details:
(i) The equipment used (e.g., HPLC system, detector, etc).
(ii) The exact duration of the analytical steps (sample analysis).
(iii) The duration of vortexing during sample preparation, as this can affect consistency.**

The methodology section was modified and summarized to be more concise:

Two tables were added:

- Table 3. HPLC stability-indicating dosing methods, general parameters
- Table 4. Vitamin capsules treatment before HPLC-UV analysis

The corresponding paragraphs were deleted (L106 to L111, L119 to L124, L132 to L136 and L144 to L148).

L102: “General parameters and vitamin capsules treatment before analysis are summarized in Tables 3 and 4.” was added.

**6- Stability Studies: Please specify the reference for the climatic chamber used in the study. It would also be beneficial to use the terms ‘stability chamber’ or ‘environmental test chamber’ instead, as these are more commonly used in the pharmaceutical field to describe equipment used for stability testing. This terminology is more precise and aligns with established industry standards.**

L205: “a climatic chamber (25˚C, 60% RH (Relative Humidity))” was replaced by “an environmental test chamber (25˚C, 60% RH (Relative Humidity)) (ICH110, Memmert)”

**Results section:
7- Unless mistaken, this manuscript nor its supporting data does not seem to include the spectra for each compound alone and not degraded (reference spectra). This is a major concern as the readers should be able to judge how the degradation process affects the spectra.**

Reference spectra were added in supporting data.

**8- Tables 3-4: some RRT are close enough to the parent peak. Which guidelines or scientifically established data/norms did the authors use to validate the suitability of their methods in this matter?**

We apologize for this oversight. During method validation, especially during forced degradation, the resolution of each degradation product’s peak is calculated, and must be higher than 1.3, as reported in documents from the FDA (for validation of pharmaceutical products methods of analysis).

L227: “The lower values reported for resolution were respectively 1.79 (peak with RRT 0.95, thiamine chlorhydrate), 2.31 (peak with RRT 1.07, riboflavin), 1.41 (peak with RRT 0.91, pyridoxine chlorhydrate) and 2.84 (peak with RRT 1.10, biotin). The four methods have good specificity, because all resolution values for degradation products were higher than 1.30.^20^” was added.

A new reference was also added:

“Food and Drug Administration, Office of Regulatory Affairs. ORA Lab Manual Vol. II - Methods, Method Verification and Validation (ORALAB. 5.4.5.). [Cited 2025 January 27]. Available from: https://www.fda.gov/media/73920/download”

**9- Table 7: include precise information on which criterion was accepted based on European or US pharmacopeia and why.**

The FDA’s guidance for method validation, recommends five parameters for SST: capacity factor, resolution, theoretical plates, tailing factor and relative standard deviation.

Specific monographs in the USP and the EP never reports limit values for these five parameters, only for one to three of them.

In routine practice, we realize SSTs with limit values based on (in order of priority): the closest USP monograph for a corresponding pharmaceutical drug, the USP monograph for the corresponding Active Pharmaceutical Ingredient, and the FDA recommended limit values.

“Moreover, a System Suitability Test (SST) was proposed for each dosing method, following the USP monographs^21-24^ if available or at least the FDA recommendation for pharmaceutical analysis.^25^” was replaced by “Moreover, a System Suitability Test (SST) was proposed for each dosing method. The limit values were based on (in order of priority, if available): the USP monograph of the corresponding pharmaceutical drug,^21-22^ the USP monograph of the corresponding Active Pharmaceutical Ingredient^23-24^ or the FDA recommendations.^25^”

Table 7 (now Table 10…) footnotes were modified, to specify the precise information from the provenance of each criterion as requested by the reviewer.

**10- Table 8 and corresponding text: please precise which criterion/assay was used to reject the batches.**

As explained before, we added in the supporting information a short summary of the criteria / assay used in routine practice to control batches conformity.

L244: “Routine quality control and results from the first three batches analyzed are summarized in the supporting information.” was added.

L247: “for failure of uniformity test (2.9.40.)” was replaced by “for failure of uniformity of dosage units test (acceptance value higher than 15.0%).”

**11- I would rather be able to see the standard deviations on each point, so maybe Figure 1 could be divided into 4 smaller figures to include this important dataset.**

We agree with the reviewer, and we apologize once again: this was already done (in supporting information, to minimize the number of figures in the main manuscript), but not clearly indicated. To be more informative, the following modification was done:

L255: “Results are summarized in Figure 1 and are detailed in supporting information (one figure per molecule, with CI95).” Was replaced by: “Results are summarized in Figure 1. This figure is also divided into 4 figures, one for each molecule, with CI95 (represented as thin lines), available in the supporting information (Figures E-H).”

**12- Figure 2: legend is not detailed enough to understand what is presented on this figure. Thin lines, thick lines? SD?**

L261: (Figure 2) was replaced by (Figure 2, CI95 represented as thin lines)

**Discussion Section:**

**13- The discussion does not address whether the HPLC methods used are novel or adapted from existing protocols, such as those outlined in pharmacopeias or previous studies.**

The first point was added to the “method” section.

L102: “was adapted from the method described in the European Pharmacopoeia (“Thiamine hydrochloride” monograph) with slight variations in the mobile phase and the gradient. It” was added

L116: “was developed based on the European Pharmacopoeia (“riboflavin” monograph) with significant modifications in the mobile phase and the mode (from gradient to isocratic). It” was added

L128: “The pyridoxine dosing method was developed from the United States Pharmacopoeia (“pyridoxine tablets” monograph, dissolution test) with modifications in the column specifications, the injection volume and the flow.” Was added

L129 “for the pyridoxine hydrochloride dosing method” was deleted

L141: “developed from the United States Pharmacopoeia (“biotin capsules” monograph) with significant modifications in the column specifications and injection volume. It was” was added

**Additionally, while degradation products are mentioned, they are only observed rather than identified.**

We completely agree with this remark, and wish we could implement it, but public hospital structures lack resources for such studies. We highlight this point as a limitation of our work in the discussion.

L319: “In the pharmaceutical industry, when degradation products are identified and characterized, their pharmacological relevance and/or their toxicity can be studied. However, during small-scale compounding’s stability studies, hospital pharmacists do not have the resources to perform such studies. This is one of the limitations of our study, but also more generally of every published stability study, attempting that the pharmaceutical industry rarely publishes its data.” was added.

**14- For some of these products, the relative retention times (RRT) are similar to that of the parent drug, suggesting that the resolution of the method could be optimized. If improving the resolution is not feasible, this limitation should be explicitly acknowledged and discussed.**

We omitted to add the lowest reported resolutions for each method. We added a short paragraph L227, as reported before, with these values. The lowest resolutions were not lower than 1.3, which is the reference value reported in the FDA’s guidance for method validation and method verification.

Moreover, both in the USP and the EP, based on our knowledge and experience, Rs limit values can be established at 1.3 or even lower in several monographs (we have never seen values lower than 1.2 in our practice). For example, in methods we routinely conduct: USP “spironolactone” monograph specifies that the Rs between spironolactone and spironolactone related compound B must be higher than 1.2; the EP “gentamicine” monograph states that the Rs between gentamicine C1a and impurity A must be higher than 1.2…. With resolutions above 1.3 (and peaks visually identified as distinct from the parent peak during forced degradation experiments), we considered it was not a limitation.

**15- The discussion section is relatively lengthy and could benefit from being more concise while ensuring the relevance of each point.**

Several paragraphs and sentences were deleted, from L285 to L287, and from L319 to L332.

**16- Given that the results section requires revision, the discussion should also be adjusted to reflect these changes.**

Modifications were done to the discussion section, according to the revision of the results section.
